# Phylogenetic evidence that both ancient vicariance and dispersal have contributed to the biogeographic patterns of anchialine cave shrimps

**DOI:** 10.1038/s41598-017-03107-y

**Published:** 2017-06-06

**Authors:** José A. Jurado-Rivera, Joan Pons, Fernando Alvarez, Alejandro Botello, William F. Humphreys, Timothy J. Page, Thomas M. Iliffe, Endre Willassen, Kenneth Meland, Carlos Juan, Damià Jaume

**Affiliations:** 1 Dept. of Biology, Universitat de les Illes Balears. Ctra. Valldemossa km 7’5, Palma, 07122 Balearic Islands Spain; 2 IMEDEA (CSIC-UIB), Mediterranean Institute for Advanced Studies. C/ Miquel Marquès 21, Esporles, 07190 Balearic Islands Spain; 3 Colección Nacional de Crustáceos, Dpto. de Zoología, Instituto de Biología, UNAM. Tercer circuito s/n, Ciudad Universitaria, Copilco, Coyoacán, A.P. 70-153, México D.F. CP, 04510 Mexico; 4 Dept. de Ciencias Químico-Biológicas, Instituto de Ciencias Biomédicas, Universidad Autónoma de Ciudad Juárez. Anillo del Pronaf y Estocolmo s/n, Ciudad Juarez, 32300 Chihuahua, Mexico; 5 Western Australian Museum, Collections and Research, Locked Bag 49, Welshpool DC, WA 6986 Australia; 6 School of Animal Biology, The University of Western Australia, Crawley, Perth, Western Australia 6009 Australia; 7 Australian Rivers Institute, Griffith University, Nathan, Queensland 4111 Australia; 8 Water Planning Ecology, Queensland Dept. of Science, Information Technology and Innovation, Dutton Park, Queensland 4102 Australia; 9 Dept. of Marine Biology, Texas A&M University at Galveston, 200 Seawolf Parkway, OCSB #251, Galveston, TX 77553 USA; 10 Dept. of Natural History, University Museum of Bergen, Postboks 7800, N-5020 Bergen, Norway; 11 University of Bergen, Department of Biology, PO Box 7800, N-5020 Bergen, Norway

## Abstract

Cave shrimps from the genera *Typhlatya*, *Stygiocaris* and *Typhlopatsa* (Atyidae) are restricted to specialised coastal subterranean habitats or nearby freshwaters and have a highly disconnected distribution (Eastern Pacific, Caribbean, Atlantic, Mediterranean, Madagascar, Australia). The combination of a wide distribution and a limited dispersal potential suggests a large-scale process has generated this geographic pattern. Tectonic plates that fragment ancestral ranges (vicariance) has often been assumed to cause this process, with the biota as passive passengers on continental blocks. The ancestors of these cave shrimps are believed to have inhabited the ancient Tethys Sea, with three particular geological events hypothesised to have led to their isolation and divergence; (1) the opening of the Atlantic Ocean, (2) the breakup of Gondwana, and (3) the closure of the Tethys Seaway. We test the relative contribution of vicariance and dispersal in the evolutionary history of this group using mitochondrial genomes to reconstruct phylogenetic and biogeographic scenarios with fossil-based calibrations. Given that the Australia/Madagascar shrimp divergence postdates the Gondwanan breakup, our results suggest both vicariance (the Atlantic opening) and dispersal. The Tethys closure appears not to have been influential, however we hypothesise that changing marine currents had an important early influence on their biogeography.

## Introduction

Some aquatic obligate cave crustacean genera (known as stygobionts) show a patchy but surprisingly broad distribution across the globe^1^. This is particularly so for species that are largely restricted to coastal aquifers affected by marine intrusion (anchialine environment), or to fresh inland ground waters, in areas formerly covered by epicontinental seas^1–3^. This pattern is found across tropical-subtropical latitudes and covers both continents and oceanic islands^1^, and is shared by a varied array of taxonomic groups (remipedes, ostracods, copepods, amphipods, isopods, mysids, thermosbaenaceans, decapods), to the point that representatives of the same genera frequently co-occur^4^. The high level of endemicity and low frequency of sympatry exhibited among congeneric species suggest their dispersal abilities are extremely limited, which makes sense given their cave-adapted lifestyle. The combination of a large distribution and presumed limited vagility suggests that this pattern could have been caused by the fragmentation of a continuous common range formerly shared by the ancestors of these taxa (vicariance)^4^.

Vicariance and dispersal are two main processes traditionally used to explain geographical distribution of organisms, and the relative contribution of each to contemporary biogeographic patterns is often contentious and complex^5, 6^. Some approaches have made an *a priori* rejection of long-distance dispersal in favour of ancient vicariant events to explain biogeographic patterns, such as the panbiogeographic method of Croizat^5, 7, 8^. This approach has in turn been criticised for not considering alternative hypotheses^9^ or incorporating phylogenetic analyses^10^. Dispersalist theories were popular in the early history of biogeography (e.g. Darwin and Wallace), and have been revived by the recent addition of molecular data^11^.

Stygobiont crustaceans from anchialine habitats thus provide an excellent case study to tease apart the roles of vicariance and dispersal in biogeographic history, given their derivation from marine ancestors and extremely disjunct distributions^12^. Vicariance via plate tectonics has usually been put forward as the main mechanism underlying their patterns^1^, in particular the opening of the Atlantic Ocean, fragmentation of Gondwana, and the closing of the Tethys Sea. In possible contrast, some of these same supposedly highly restricted taxa have life-history traits that should promote dispersal, such as highly salt tolerant larvae^13^.

**Figure 1:**
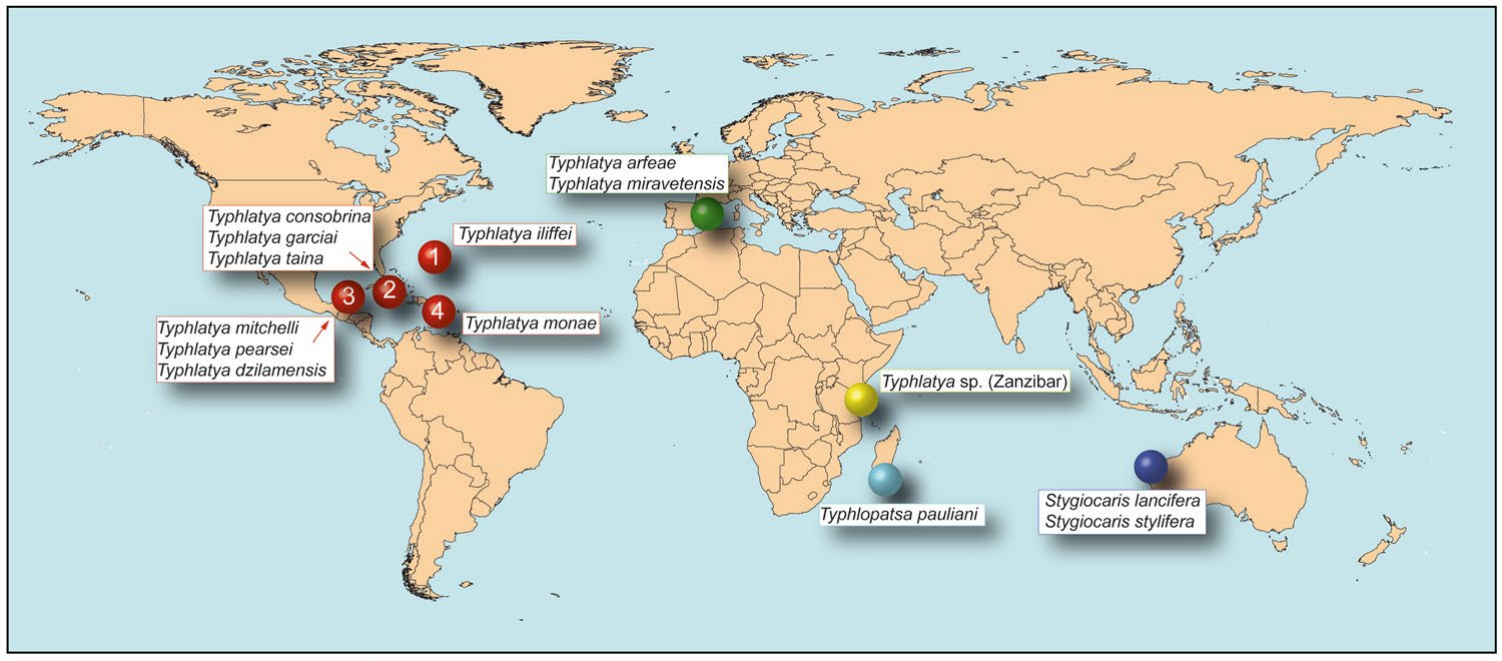
Distribution of the sampled taxa in the TST complex of anchialine cave shrimps (Atyidae). Map adapted from https://en.wikipedia.org/wiki/File:BlankMap-World6-Equirectangular.svg under a Creative Commons CC0 1.0 Universal Public Domain Dedication. Full terms at: https://creativecommons.org/publicdomain/zero/1.0/deed.en. Colours of circles are used in Figs 2 and 4 for biogeographic considerations.

Here we attempt to distinguish between long-distance dispersal and vicariance as factors accounting for the extremely disjunct contemporary distribution in a shrimp group spread across the globe, the *Typhlatya*/ *Stygiocaris*/ *Typhlopatsa* (TST) complex^14^, within the family Atyidae. This complex comprises twenty cave-adapted (troglomorphic) taxa, which occur in the Caribbean, Atlantic (Bermuda & Ascension islands), western Mediterranean and Indian Ocean (*Typhlatya*; 18 spp. including an undescribed species from Zanzibar); northwestern Australia (*Stygiocaris*; 2 described spp.) and Madagascar (*Typhlopatsa*; 1 sp.) (Fig. 1). However, the TST complex does not include the *Typhlatya* species from Galápagos (*T. galapagensis*), that belongs to a different lineage^15^. Most of these shrimp species are anchialine, except *Typhlatya campecheae* and *T. miravetensis*, which live only in fresh inland ground waters. None of the species has ever been found in open marine habitats, and most are known only from a single island or narrow portion of coast^15^.

The biogeographic history of the TST shrimp-complex is particularly puzzling since there is no simple way to reconcile its apparent confinement to the highly specialised subterranean water environment with its occurrence in areas separated by broad and deep oceanic basins or entire continental landmasses^14^. As mentioned above, a vicariant hypothesis regarding how such an extreme disjunct distribution was generated assumes that the group derives from a broadly distributed shallow-water late Mesozoic –Tethyan– marine ancestor^1, 14, 16^. The fragmentation of its primary range by plate tectonics (i.e. opening of deep oceanic basins, blocking of former marine seaways by displaced continental landmasses and disruption or shift of marine currents) led to isolation of the populations that ultimately gave rise to the present cave species. The presence of representatives on geologically young oceanic islands could be explained if these islands had existed as shallow banks or seamounts since before the complete disruption of the Tethys Sea, so that the ancestors of the recent species could remain associated with these marine shallow waters until the emergence of the islands^17^.

Vicariance via the fragmentation of the Tethys Sea has been postulated because of congruent divergence estimates in the phylogenetic analyses of other groups of stygobiont crustaceans with a similar distribution to the TST shrimp-complex, such as the amphipod family Metracrangonyctidae^18, 19^. Similarly, a recent study^20^ on anchialine fishes (*Milyeringa*/*Typhleotris*), that inhabit the very same caves in NW Australia and Madagascar as the *Stygiocaris*/*Typhlopatsa* shrimp considered here, has suggested vicariance by fragmentation of Gondwana as the mechanism to explain their sister-group relationship. However, the inference of ancient divergences can be problematic, as evidenced by the aforementioned studies^21, 22^, which highlight some methodological issues, in particular alternative fossil calibrations, which can yield younger estimates and thus imply dispersal.

Although adult atyids have never been reported from open marine habitats, it is well established that some members of the family require saline water for larval development^13, 23^, which suggests a way in which seemingly unlikely oceanic dispersal could be possible. Anchialine cave water provides a salinity gradient interphase between fresh ground water and marine water and may offer suitable, vertically segregated, environmental conditions for both adults and juveniles^24^. Some anchialine species, including other atyids, occur on oceanic islands thousands of kilometers apart, sometimes with the same mitochondrial haplotypes^15, 25–27^. The divergent phylogeographic patterns exhibited among anchialine shrimp have been linked to differences in the duration of their respective planktonic larval (dispersive) phases^26, 28^. However, it is the planktonic characteristics of ancestral populations from millions of years ago, not contemporary ones, that may have generated some of these patterns, and so all we can do is infer and hypothesise. Even so, the fact of a wide current distribution suggests that trans-oceanic dispersal could also have played an important role in the generation of the extreme disjunct pattern exhibited by the TST shrimp-complex.

Previous phylogenetic studies on the TST shrimp-complex have relied on a limited number of species and on relatively short DNA sequences^15, 29–32^. These studies were inconclusive with regard to the causes and time-frame of the extreme disjunct distribution of the complex. The most comprehensive of these studies^15^ was based on mitochondrial and nuclear DNA sequences from representatives of distinct areas, but did not include the Malagasy *Typhlopatsa* ^33^ or the newly discovered Zanzibari *Typhlatya*, and thus could not consider Gondwanan vicariance. The inferred molecular phylogeny was calibrated by constraining three nodes based on palaeogeographic considerations, which provided node age estimates that were inconsistent with a simple vicariant scenario by plate tectonics^15^.

Here we have taken a whole mitochondrial genome approach to resolve phylogenetic and biogeographic relationships by sequencing fourteen species from three genera from the TST complex that represent all of the geographic regions where it is known to occur. In particular, we test the ancient vicariant origin hypothesis, which if true would be supported by the following predictions: (1) divergence times of sister-species on the two shores of the Atlantic Ocean should be compatible with the opening of this oceanic basin (ca. 110 Ma^34, 35^); (2) divergence times between sister lineages on opposite sides of the Indian Ocean should be consistent with the establishment of deep-water conditions associated with the separation of the Australian and the Indo-Madagascar landmasses of Gondwana (that started at 132 Ma^36^); and (3) the divergence of Indian Ocean and Mediterranean taxa should trace back to the occlusion of the Tethys sea (20–16 Ma^37^). We assess these predictions using fossil-calibrated phylogenetic trees, together with lineage diversification analyses, to appraise the potential roles played by both vicariance and long distance dispersal in the biogeographic history of this group.

## Results

### Molecular phylogenetics

Sixteen new mitogenomes were produced (Table 1). All sequenced mitogenomes matched the ancestral putative pancrustacean gene order and included 13 protein-coding genes (PCGs), 22 transfer RNAs (tRNAs) and two ribosomal genes. The final concatenated alignment of the 13 mitochondrial PCGs for the 30 studied taxa (outgroups included) comprised 10,953 bp (Supplementary Table 1). Xia’s^38^ test showed low levels of substitution saturation even under the assumption of an extremely asymmetric tree (ISS = 0.405, ISSc = 0.818, *P* two-tailed <0.00001). Similar results were found when testing for saturation at each codon position separately, except for third coding positions where the occurrence of significant levels of saturation was detected under the hypothesis of an extremely asymmetric tree (ISS = 0.741, ISSc = 0.554, *P* two-tailed <0.00001). However, the balance of the phylogenetic tree obtained under the best partitioning scheme (see below) was TCI = 1414 from a set of possible values ranging from 338 to 3654, thus suggesting that our phylogeny is far from being asymmetrical. The best partitioning scheme for the nucleotide-based analyses consisted of subdividing the dataset by codon position and coding DNA strand (Supplementary Tables 2 and 3). Other partitioning strategies analysed resulted in an identical topology (Supplementary Fig. 1a–p) except for the single partition scheme (Supplementary Table 2), which differed in the phylogenetic placement of *Typhlatya garciai* and *T. pearse*i, although this partition scheme was the worst ranked based on the Bayesian Information Criterion (BIC). Codon-based substitution models, exclusion of the third coding positions or protein data resulted in the same topology or topologies showing minor differences (Supplementary Fig. 1h–m and 1p). Phylogenies based on single genes were both less resolved and supported than the tree based on the 13 PCGs (see Supplementary Fig. 1q for the tree obtained with the widely used cytochrome oxidase subunit 1 marker as an example).

**Figure 2:**
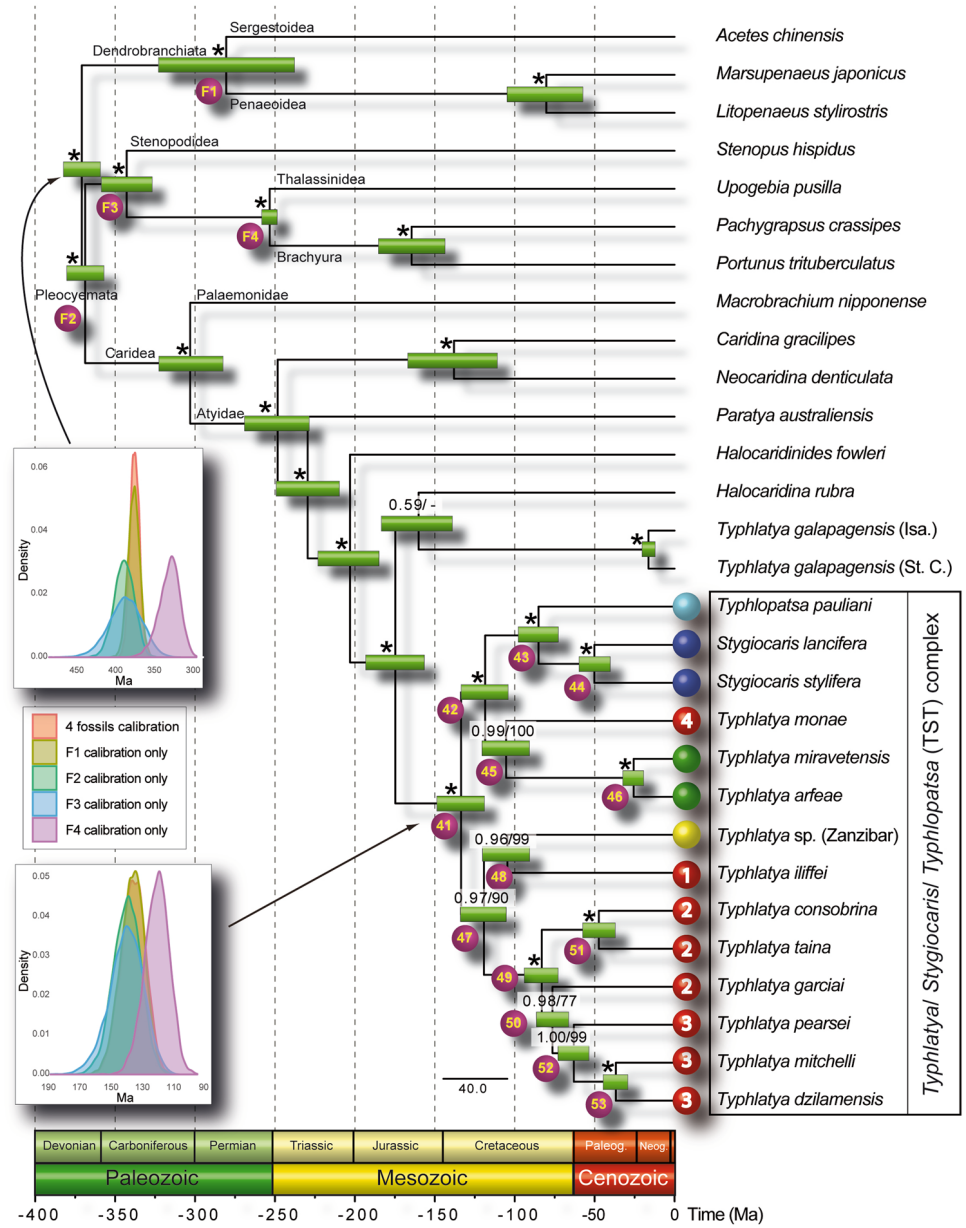
Dated phylogeny of the TST complex and outgroup taxa based on nucleotide sequences of the 13 protein-coding mitochondrial genes. Numbers above nodes indicate support values (Bayesian posterior probability/ML bootstrap support). Nodes with maximum nodal support (i.e. 1.00/100) are marked with asterisks. Nodes defining the TST phylogeny are numbered for discussion in the main text (nodes 41 to 53). Fossil calibration points are indicated with codes F1 to F4. Inset multi-plot charts show the age density distributions for two relevant nodes (the root of the tree and the origin of the TST clade) resulting from analysing the data set using each single-fossil separately or all-fossils-at-a-time as calibration points. Coloured circles on the left of the TST complex species names refer to their respective geographical distributions according to the map on Fig. 1.

The inferred tree was fully congruent with most recent phylo-mitogenomic proposals on decapod systematics^39^ and relationships amongst atyid taxa^32^ (Fig. 2). The TST complex was consistently recovered as a monophyletic lineage with maximum nodal support (Fig. 2 – node 41), rendering the genus *Typhlatya* paraphyletic as it currently stands since both *Stygiocaris* and *Typhlopatsa* nest within it. Within the TST clade, two well-supported monophyletic sub-lineages occur (Fig. 2). One (node 42) includes the Australian genus *Stygiocaris* and the Malagasy *Typhlopatsa*, the Mediterranean species *T. arfeae* and *T. miravetensis*, and the Caribbean species *T. monae*. The analysis recovers *Stygiocaris* and *Typhlopatsa* as sister-groups with maximum support, and the same holds for the two Mediterranean *Typhlatya* species. The phylogenetic relationships within the other sub-lineage, which includes *Typhlatya* species from the western Atlantic (Mexico, Cuba, Bermuda) and eastern Africa (Zanzibar) (node 49), were also identical in all the analyses performed except for the unsupported relative position of the Cuban *T. garciai* in the protein analysis based on the CAT profile mixture model^40^. The systematic position of *T. iliffei* (Bermuda) and *Typhlatya* sp. (Zanzibar) could not be established with confidence within this sub-lineage.

### Divergence time and diversification

The best fitting clock model resulted from setting an independent relaxed log-normal clock model for each partition plus a Yule diversification pattern (Supplementary Table 4). Node ages and their respective 95% highest posterior densities (HPD95%) are shown in Supplementary Table 5 for the analyses with the best Bayes factors using fossil calibrations. The four fossil calibration points (Supplementary Text 1) showed age congruence with each other along the phylogeny as deduced from the cross validation test (Supplementary Fig. 2, and Fig. 2 for the age density distributions in the root of the tree and for the origin of the TST clade), but were largely incompatible with biogeographic calibrations^15^. The latter rendered much younger node ages and unexpectedly high mitochondrial substitution rates, and their combination with fossil data in the same analysis resulted in a high variation in the substitution rates across branches (i.e. high ucld.stdev values) (Supplementary Figs 3–5).

The selected chronogram that used only fossil constraints placed the origin of the TST complex in the early Cretaceous (133.6 Ma, HPD95% = 148.9–118.8; Fig. 2 – node 41). Within the TST complex, two main ancestral lineages were both inferred to date to relatively soon afterwards, with the western Atlantic/east African lineage (node 47) first diverging 119.2 Ma (133.9–105.2 Ma) and the Australian/ Malagasy/ Caribbean lineage (node 42) 118.5 Ma (133.7–104 Ma). The divergence between the Australian *Stygiocaris* and the Malagasy *Typhlopatsa* (node 43) was dated to later in the Cretaceous at 85.1 Ma (97.9–72.7 Ma), while the split between the Caribbean species *T. monae* and the Mediterranean species *T. arfeae* and *T. miravetensis* (node 45) is estimated at 105.4 Ma (120.9–90.6 Ma). When 3^rd^ codon positions were removed from the dataset, the age for node 43 (Australia/Madagascar) was inferred as 70.0 Ma (81.8–58.4 Ma), and node 45 (Caribbean/Mediterranean) as 82.3 Ma (95.7–68.9 Ma) (Supplementary Table 5).

**Figure 3:**
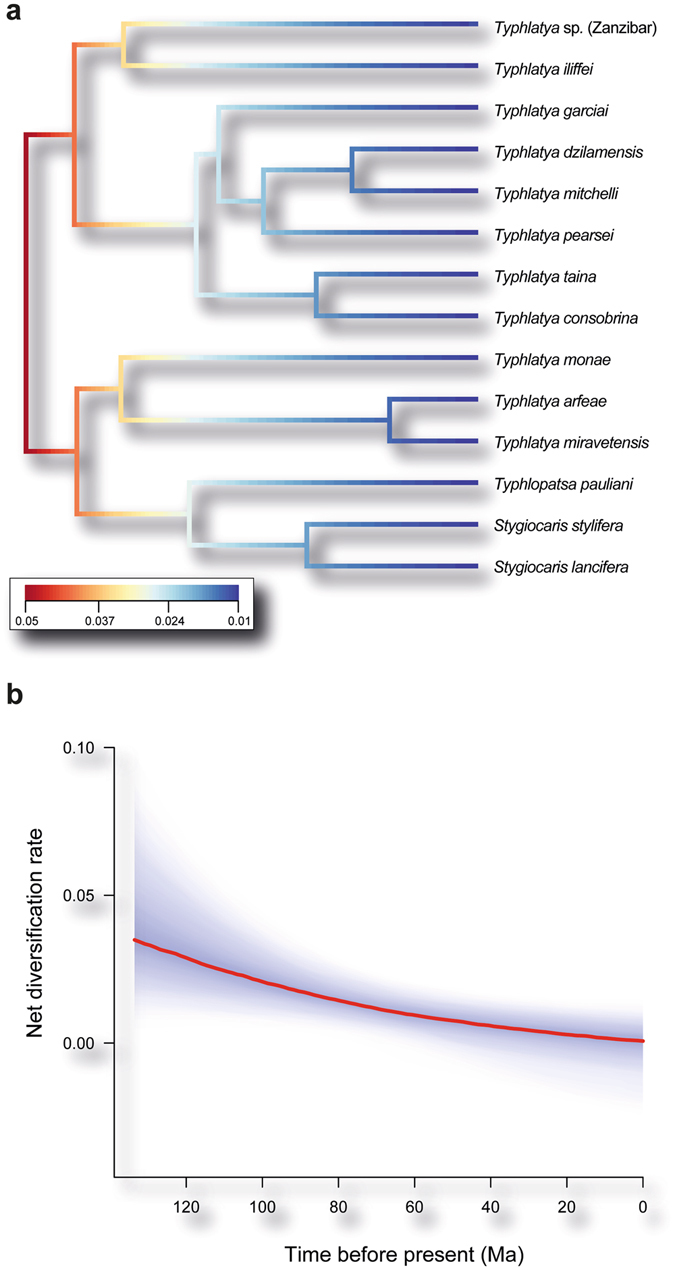
(**a**) Ultrametric tree showing speciation rates (cool colours = slow, warm = fast) along each branch of the TST complex. Each unique colour section of a branch represents the mean of the marginal posterior density of speciation rates on a localised segment of the tree. Note the temporal deceleration in speciation rates toward tips (warm colours at the root, cool colours at the tips). (**b**) Evolutionary net diversification rates through time (i.e., speciation minus extinction rates). Colour density shading denotes the 10% through 90% Bayesian credible regions on the distribution of rates at any time.

Diversification took place early in the TST complex as inferred from the significantly negative gamma value, thus rejecting the null hypothesis of a constant diversification rate (γ = –2.71, *P* one-tailed = 0.003, *P* two-tailed = 0.007). The same result was obtained when correcting for incomplete taxon sampling (critical γ value = –1.9; *P* = 0.008) and using the lineages-through-time (LTT) plot (Supplementary Fig. 6). Accordingly, both LASER^41^ and DDD^42^ analyses independently resulted in the selection of a diversity-dependent diversification process as the best fitting model for our dataset (Supplementary Tables 6 and 7). Moreover, BAMM^43^ analyses revealed that the diversification rate has progressively decreased over time without causing any significant shift in the evolutionary history of the TST complex (posterior probability = 0.92; Fig. 3a). These results are consistent with mean speciation and extinction rates defining a low net diversification rate for this lineage of cave shrimp (0.0088 events/Ma/lineage) (Fig. 3b).

### Biogeographical history of the TST complex

**Figure 4:**
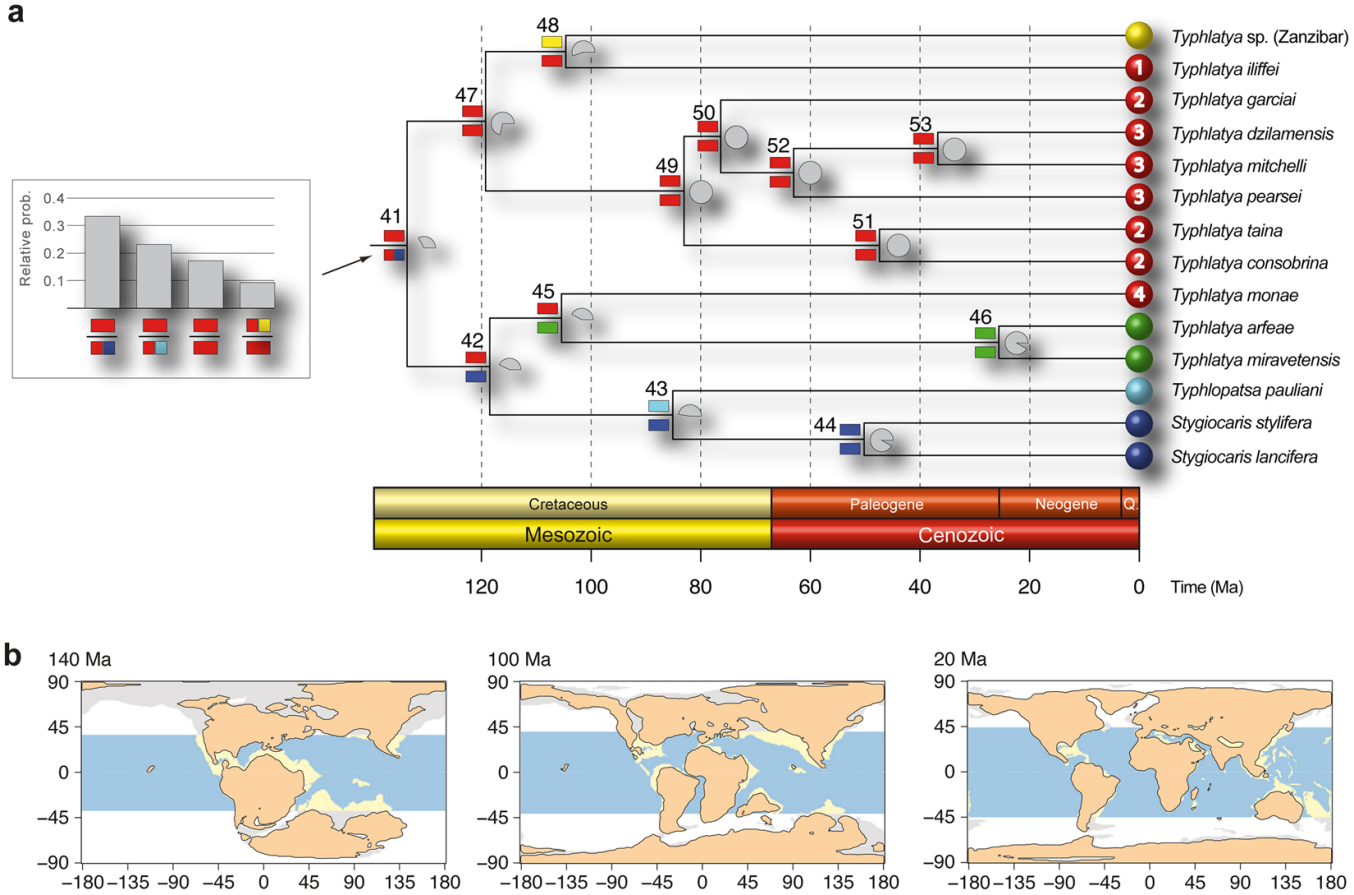
(**a**) Reconstruction of the biogeographic history of the TST complex inferred in LAGRANGE. Colours defining the distribution areas refer to the map in Fig. 1. Coloured rectangles on the left of each node show the most probable inherited ranges for each of the two daughter branches (see Supplementary Table 8 for a complete report on the relative probabilities for all the splits). Grey pie charts on the right of each node reflect the relative probability of occurrence of that split. Histogram on the left of the tree depicts the four most probable biogeographic episodes for the origin of the TST clade. Nodes are numbered according to Fig. 2 for their discussion in the main text. (**b**) Distribution of shallow and deep-ocean sea floor across the past 140, 100 and 20 Ma. Blue colour represents deep tropical ocean, yellow represents tropical shallow reefs. Maps adapted from Leprieur *et al*.^46^ (http://www.nature.com/articles/ncomms11461/figures/1) under a Creative Commons Attribution 4.0 International License. Full terms at: https://creativecommons.org/licenses/by/4.0/.

LAGRANGE analyses based on three matrices of migration probabilities resulted in similar log-likelihood values: -lnL = 19.24, 19.69 and 21.49 for three, two and a single time intervals, respectively. Hence, we selected the three and two time slices models following the standard cut-off value of two log-likelihood units difference^44^. Both models resulted in the same hypothesis regarding historical biogeography (Fig. 4). Thus, the most recent common ancestor (mrca) of the TST complex (node 41) was inferred to live along the western shores of Tethys, in the area corresponding to the Proto-Caribbean and the Proto-Atlantic, and there it diverged into two primary lineages (Supplementary Table 8). One of them (node 47) remained in the same ancestral area except for the lineage leading to the current Zanzibari species (node 48). Some members of the second main lineage (node 42) remained in the Proto-Caribbean (*T. monae*), but others became established in what is now the W Mediterranean (node 45) and Indian Ocean (node 43). In both cases the very disjunct distributions within the two lineages (nodes 45 and 48) occurred very early in the diversification of the genus (mid-Cretaceous), before the regional groups of species formed. S-DIVA analyses implied the same biogeographical hypotheses as LAGRANGE (Supplementary Fig. 7) and allowed for hypotheses of nodes in the phylogeny where dispersal and/or vicariance events likely took place. Dispersal was inferred to have occurred in the mrca of the TST clade (node 41) and also in the more weakly supported node 47, while vicariance episodes were inferred for nodes 42, 43, 45, and 48.

## Discussion

Our mitogenomic analyses show the TST complex is divided into two main ancestral lineages. A strongly supported clade (node 42) comprises the taxa from NW Australia and Madagascar, the W Mediterranean and one of the species from the Caribbean (*T. monae)*. The other lineage (node 47) contains species from the likely ancestral area of the western Atlantic (Yucatan Peninsula and Cuba, plus Bermuda), as well as an undescribed new species from east Africa (Zanzibar).

The first plate tectonic vicariant scenario of particular interest in TST’s evolutionary history relates to sister-species on opposite shores of the Atlantic, and whether this aligns with the establishment of deep-water conditions in the opening Atlantic at 110 Ma^34, 35^. The two main lineages present in our phylogeny (nodes 42 and 47) were inferred to date back to the early Cretaceous (148.9–118.8 Ma), a period when the Tethyan Seaway was at its largest. Our estimated time frame for the split between the species separated by the Atlantic Ocean in both ancestral major lineages is compatible with the age of the first establishment of deep-water conditions between the east and west shores of this oceanic basin^34, 35^. Thereby, the separation of the lineages currently present on Bermuda and Zanzibar (node 48 in Fig. 2) was estimated to occur at 121–91 Ma, and the split between *T. monae* (Caribbean) and the western Mediterranean species (*T. arfeae*, *T. miravetensis*) (node 45 in Fig. 2) also falls within the same age range, suggesting that the two splits may have been triggered by the disconnection between the east and west Atlantic shores, and thus relate to vicariance via tectonic movements.

The second vicariant scenario on which our phylogenetic data may be informative is that associated with the separation of the Australian and the Indo-Madagascar landmasses of Gondwana. Our phylogenetic analysis recovers a strongly supported sister relationship between the NW Australian *Stygiocaris* and the monotypic, SW Malagasy genus *Typhlopatsa*. Both taxa are strictly subterranean and are known to occur only in anchialine karstic caves separated by a 7,000 km span of deep-ocean. Interestingly, the two genera were considered to be closely related by their original descriptor^45^. Moreover, in a previous study using nuclear and mitochondrial markers from numerous surface and cave-dwelling atyids from the Indo-Pacific (but not *Typhlopatsa*), *Typhlatya* was recovered as sister to *Stygiocaris* ^31^.

The chronogram with the best score suggests that the Australian-Malagasy clade (node 43) is Late Cretaceous in age (85.1 Ma; 97.9–72.7 Ma). At that time, Antarctica/Australia was already well separated from the Indo-Madagascar landmass as part of the sequential break-up of the supercontinent Gondwana. The estimated age for the split between the Australian and the Malagasy shrimp genera would invalidate a vicariant origin by fragmentation of Gondwana as this would postdate the disconnection of the shallow continental shelf waters by 30–35 Ma on average^36, 46^, suggesting instead an origin associated with ancient trans-oceanic dispersal. Recently an ancient vicariant origin by plate tectonics was proposed by Chakrabarty *et al*.^20^ to explain the sister relationship found between the obligate cave-dwelling gobiid fishes *Milyeringa* (NW Australia) and *Typhleotris* (Madagascar), which mirror exactly the distribution displayed by the Australian-Malagasy shrimp clade. However reanalyses of this relationship suggests that it also postdates any continental connection by tens of millions of years^22^. Interestingly our S-DIVA analysis inferred vicariance for the same node, but this algorithm has been shown to favour vicariance over early dispersal^47^. In any event, a *recent* long-distance trans-oceanic dispersal is an unlikely explanation for the Australoid-Malagasy shrimp or fish relationship given the deep genetic divergence between the sister lineages.

The third geologically derived vicariant event that we have considered is the final occlusion of Tethys by the collision of the Arabic and the Anatolian Plates at ca. 20–16 Ma^48^. This scenario is different from the previous two, in which the developing barriers which may have sundered populations were represented by expanding deep waters. In this case, the barrier is represented by land as the eastern Mediterranean was blocked from the Arabian Sea. Our age estimates for the divergences between the species from the Indian Ocean (Zanzibar, Madagascar, W Australia) and the rest of taxa (western Atlantic, Mediterranean) exceeds the date established elsewhere for the occlusion of the connection between the Mediterranean and the Indian Ocean (20–16 Ma^37^) by at least 70 million years (nodes 42, 48). This strongly implies these divergences are unrelated to the closure of the Tethyan Seaway.

However, our deep estimates for divergences at either side of the Tethyan Seaway broadly overlap (node 42: Caribbean/Mediterranean vs Madagascar/Australia 134–104 Ma; node 48: Bermuda vs Zanzibar 121–91 Ma). This suggests that these divergences could have been caused by the same historical biogeographic event, but one much more ancient than the closure of the Tethyan Seaway. One possibility is the early Cretaceous disruption of the circum-equatorial west-flowing current system established through the Tethys since its opening in the Late Jurassic times^49^. This disruption was mainly due to the establishment of new connections of the Tethys to other oceanic basins, plus the broadening of those formerly established. We suggest that this might be the basis of the differentiation that took place within the two major lineages of the TST shrimp complex.

The calibration of molecular clocks and their application to infer the biogeographic history of evolutionary lineages are often contentious and complex^22^. Molecular clock calibrations are usually enforced by assigning well-dated and confidently identified fossils to particular nodes of phylogenies^50^. Node age estimates in our TST phylogeny were explored using four decapod fossils as calibration priors and compared with the results obtained using three geological events hypothesised to have had biogeographic influence on contemporaneous TST populations. Fossil ages were largely congruent in cross validation analyses, which has not always been the case for atyid time-trees^32^. However they were largely incompatible with biogeographic-based geological calibrations (Supplementary Figs 3–5). It has been argued that calibrations solely based on geological events can hinder biogeographic hypotheses as they can lend to a ´circular reasoning by presupposing the very speciation mode they are trying to test´^51^, while geological information is advised to be assessed independently of a paleontological one^52^. In addition, calibrations near the root of phylogenetic trees are important, although deep calibrations can lead to spurious results when inferring the age of relatively shallow nodes^53^. Therefore, we have favoured the estimates based on fossil calibrations, at least for deeper nodes not close to the tips of the tree.

Our results on species diversification show that the ancestors of the current species in the TST complex diversified early in the phylogeny. This was followed by a gradual slowdown in diversification in a diversity-dependent manner. The latter might be related to a life-style transition from the open sea to the highly specialised anchialine and subterranean fresh water environments. This is a process presumably associated with the progressive reduction in dispersal abilities^15^. Overall, the troglomorphic adaptations to the subterranean habitat may have ultimately driven these species to become virtually landlocked in coastal aquifers, separated by broad oceanic basins^15^. Accordingly, for a formal reconstruction of the biogeographic history of the TST complex we made the assumption that the ancestral shrimp lineages populated – either as adults, larvae or both – a shallow tropical sea that progressively broke-up. We conservatively modelled migration probabilities among shallow waters according to historical plate tectonic information^46^. The pattern obtained suggests the existence of an ancestral shrimp lineage distributed along the Proto-Caribbean and Proto-Atlantic (node 41). This ancestor diverged into the two above-mentioned main lineages, with one remaining largely tied to this ancestral area, except for the branch leading to the species from Zanzibar. The other main lineage resulted in a broad distribution that encompassed not only the Proto-Caribbean, but also the current W Mediterranean and Indian Ocean basins, with implications of both dispersal and vicariance.

In conclusion, we examined the influence of vicariance and dispersal on the evolutionary history of a group of anchialine shrimp through phylo-mitogenomics, biogeographic/diversification analyses and calibration of molecular clocks. Our results reflect the complexity of inferring very ancient, widespread events, since they are distant in time, and are unlikely to be the result of a single, simple process. We found evidence for both major processes in the biogeographic patterns evident in this group. Vicariance via tectonic forces is our current best explanation for the divergence of sister-species separated by the Atlantic, whereas ancient long distance dispersal seems more likely to explain sister-species separated by the Indian Ocean. Further, while we did not find evidence of vicariance due the influence of the relatively recent closure of the Tethyan Seaway, we may have uncovered a more ancient vicariant event. This may be the disruption or shift of marine currents in ancient oceans. The combination of these geological and biological processes may explain the fascinatingly complex patterns of isolation on distant regions exhibited by this group of cave shrimp.

## Methods

### Taxon sampling

We sequenced the mitogenomes of 14 of the 20 species in the TST complex, plus the mitogenomes of the related *Halocaridines fowleri* and two specimens of *T. galapagensis* from different islands of the Galapagos. We also included one existing TST mitogenome (*T. miravetensis* ^54^), and retrieved the mitogenomes of twelve decapod genera and an euphausiid from GenBank to be used as more divergent outgroups (Table 1). Selection of outgroup taxa was based mainly upon the existence of fossils identified with high confidence and to assure a reasonably broad age coverage.

**Table 1:** Taxa included in the analysis, with location and GenBank sequence information.

Taxonomy	Species	Locality	Accession #
Order EUPHAUSIACEA	*Euphausia pacifica* Hansen, 1911		NC_016184
Order DECAPODA			
“Natantia”			
Suborder Dendrobranchiata			
Superfamily Penaeoidea	*Litopenaeus stylirostris* (Stimpson, 1871)		NC_012060
	*Marsupenaeus japonicus* (Spence Bate, 1888)		AP006346
Superfamily Sergestoidea	*Acetes chinensis* Hansen, 1919		NC_017600
Suborder Pleocyemata			
Infraorder Caridea			
Family Atyidae	*Typhlatya arfeae* Jaume & Bréhier, 2005	France	KX844721
	*Typhlatya consobrina* Botosaneanu & Holthuis, 1970	Cuba	KX844717
	*Typhlatya dzilamensis* Alvarez, Iliffe & Villalobos, 2005	Mexico	KX844719
	*Typhlatya galapagensis* Monod & Cals, 1970	Galápagos Is. (Isabela)	KX844711
	*Typhlatya galapagensis* Monod & Cals, 1970	Galápagos Is. (Sta. Cruz)	KX844718
	*Typhlatya garciai* Chace, 1942	Cuba	KX844720
	*Typhlatya iliffei* Hart & Manning, 1981	Bermuda	KX844710
	*Typhlatya miravetensis* Sanz & Platvoet, 1995	Spain	LT608343
	*Typhlatya mitchelli* Hobbs & Hobbs, 1976	Mexico	KX844712
	*Typhlatya monae* Chace, 1942	Dominican Rep.	KX844715
	*Typhlatya pearsei* Creaser, 1936	Mexico	KX844709
	*Typhlatya taina* Estrada & Gómez, 1987	Cuba	KX844708
	*Typhlatya* sp.	Zanzibar	KX844713
	*Stygiocaris lancifera* Holthuis, 1960	NW Australia	KX844714
	*Stygiocaris stylifera* Holthuis, 1960	NW Australia	KX844722
	*Typhlopatsa pauliani* Holthuis, 1956	Madagascar	KX844716
	*Halocaridina rubra* Holthuis, 1963	Hawaii	NC_008413
	*Halocaridinides fowleri* Gordon & Monod, 1968	Zanzibar	KX844723
	*Neocaridina denticulata* (De Haan, 1844)		NC_023823
	*Caridina gracilipes* De Man, 1892		NC_024751
	*Paratya australiensis* Kemp, 1917		NC_027603
Family Palaemonidae	*Macrobrachium nipponense* (De Haan, 1849)		NC_015073
“Reptantia”			
Infraorder Stenopodidea	*Stenopus hispidus* (Olivier, 1811)		NC_018097
Infraorder Gebiidea	*Upogebia pusilla* (Petagna, 1792)		NC_020023
Infraorder Brachyura			
Superfamily Grapsoidea	*Pachygrapsus crassipes* Randall, 1840		NC_021754
Superfamily Portunoidea	*Portunus trituberculatus* (Miers, 1876)		AB093006

Classification based on De Grave (2009). Underlined accession numbers refer to the mitogenomes obtained in the present study.

### DNA purification and mitogenome sequencing

Total DNA was purified and mitogenomes were amplified in large amplicons through long-range PCR as described in Pons *et al*.^55^. Amplicons were sequenced using a Junior NGS 454 platform and assembled into contigs in CodonCode Aligner (CodonCode Corporation, Dedham, MA, USA). Gene annotation was performed with DOGMA^56^ and MITOS^57^ WebServers.

### Phylogenetic analyses

The aligned dataset comprised the nucleotide sequences of the 13 protein-coding mitochondrial genes (PCGs). Nucleotide substitution saturation was assessed using the Xia’s test^38^. The balance of the phylogenetic tree obtained under the best partitioning scheme was measured using the total cophenetic index^58^. PartitionFinder^59^ was used to explore the partitioning schemes and evolutionary models for the standard DNA-based and the protein-based analyses. Tree topologies were analysed using maximum likelihood (ML) in IQTREE multicore v. 1.3.12^60^ specifying 1000 bootstrap replicates, and compared using BIC. We also explored the effect of excluding third coding positions from the aligned DNA matrix and the implementation of codon-based substitution models^61^, and analysing them at the protein level. In addition, the Bayesian CAT profile mixture model^40^ was also explored. Phylogenetic trees were rooted with the euphausiid included in the dataset as an outgroup.

### Estimation of divergence times

Molecular dating analyses were performed on the nucleotide sequence dataset after removing the divergent euphausiid outgroup. Tree topology, model parameter values and node ages were co-estimated and optimised in BEAST v. 2.3.2^62^. Bayesian analyses were run for 300 million generations. Mean values and confidence intervals of parameters and ages were estimated using TreeAnnotator in the BEAST package. Node ages were estimated under different clock models such as strict clock, uncorrelated log-normal clock and random local clocks (RLC). Analyses implementing RLC were run for 500–1000 million generations with the first 250 million discarded as burnin. For RLC, we implemented narrower priors as specified in Supplementary Table 9. The ages of four fossils were implemented as constraints to calibrate the relaxed molecular clock. Each calibration point was specified as a log-normal distribution where the limits of its 95% confidence interval was defined by the maximum and the minimum fossil age (Supplementary Text 1 for detailed information); namely: the oldest known dendrobranchiate shrimp (the penaeid *Aciculopoda mapesi*; Late Devonian/Famennian 374.5–359.2 Ma), the oldest known decapod *Palaeopalaemon newberryi* (Famennian 382.7–358.9 Ma); the oldest representative of the Stenepodidea (*Devonostenopus pennsylvaniensis*; Late Devonian Carboniferous-Mississippian 358.9–323.2 Ma) and finally the presumed oldest Gebiidea (*Upogebia obscura*; Early Triassic 252.17–247.2 Ma). In order to test congruence among fossil calibration points, we performed a cross validation analysis, i.e. comparing four additional BEAST runs, each calibrated with a single fossil. Alternatively, we ran the same analyses but, instead of the above four fossil age constraint priors, used three younger biogeographical events assumed to have affected the diversification of particular TST shrimp lineages as described in Botello *et al*.^15^ (Supplementary Text 1).

Different combinations of partition schemes and clock models were compared based on Bayes Factors calculated with marginal likelihood values estimated from path-sampling analyses in BEAST (40 steps with 10 million generations each). The final state files from regular BEAST analyses were used as the starting state of each step to speed up calculations. RLC analyses failed to converge even when performing steps of 50 million generations and stronger priors. Hence, we also compared models and partitioning schemes using Akaike’s information criterion (AIC) through MCMC as implemented in Tracer (AICM)^63^.

### Lineage diversification analyses

Analyses were performed on the selected BEAST dated tree. Deviations from a constant rate pure birth process were tested in the TST complex by calculating the gamma statistic^64^ using LASER v. 2.3^41^. The MCCR test^64^ was implemented to account for incomplete taxon sampling performing 500 simulations. LASER was also used to compare the fit of various models of diversification with constant and variable diversification rates using AIC. A LTT plot was generated using the R package APE 3.0^65^. BAMM v. 2.0^43^ and BAMMtools^66^ were used to estimate branch-specific rates of speciation and extinction and to determine the occurrence of distinct evolutionary processes in the phylogeny, with a global sampling fraction of 0.74. We also investigated diversification patterns using the likelihood-based method implemented in the R package DDD^42^, with parameters estimated in BAMM.

### Analysis of historical biogeography

The biogeographic history of the TST complex was explored under the dispersal-extinction-cladogenesis model (DEC) in LAGRANGE^67^, and the statistical dispersal-vicariance analysis (S-DIVA)^68^ implemented in RASP 3.2^47^. Outgroup taxa were excluded in the preferred BEAST chronogram. We constructed a biogeographic model consisting of coding terminals as occurring in any of the following five areas: Australia (A), Caribbean (including Bermuda) (C), Europe (E), Madagascar (M) and Zanzibar (Z). Migration probabilities were defined to model the changes in connectivity among marine shallow waters as described in Leprieur *et al*.^46^, with no restriction on the number of regions in which a lineage may be present. Probability values ranged from 0.1 for well-isolated areas to 1.0 for contiguous shallow waters based on historical plate tectonic processes (Supplementary Table 10). The LAGRANGE online configuration tool^67^ was used to design the migration probability matrices in different time lapses. In order to find the best-fitting time slice model, we calculated the global maximum likelihood in models with three (134–110, 110–20, and 20 Ma to the present), two (134–110 and 110 Ma to the present), and only one time interval (134 Ma to the present). A cut-off value of two log-likelihood units was applied to determine the existence of significant differences between models^44^. The same five areas scheme were used in S-DIVA analyses, accounting for phylogenetic uncertainty by performing the analyses on 1000 random post-burnin BEAST trees.

## Electronic supplementary material

Supplementary Information
