# Supplementary material for: Phylogenetic evidence that both ancient vicariance and dispersal have contributed to the biogeographic patterns of anchialine cave shrimps
Source: Sci Rep. 2017 Jun 6;7:2852. doi: 10.1038/s41598-017-03107-y (PMC5460120; doi:10.1038/s41598-017-03107-y)
Supplement: Supplementary file 1 — Supplementary Information [file 41598_2017_3107_MOESM1_ESM.pdf]

Phylogenetic evidence that both ancient vicariance and dispersal have contributed to the biogeographic patterns of anchialine cave shrimps

José A. Jurado-Rivera, Joan Pons, Fernando Alvarez, Alejandro Botello, William F. Humphreys, Timothy J. Page, Thomas M. Iliffe, Endre Willassen, Kenneth Meland, Carlos Juan & Damià Jaume

Supplementary Text 1

Tree calibration based on fossil taxa

Placement of shrimp-like decapods fossils in phylogenies of extant forms is contentious, especially when we deal with specimens with poorly calcified exoskeleton. Identification of decapod fossils usually relies on very simple features, far from the intricacies of the differential diagnoses of living taxa, which are largely based on rarely fossilized structures such as mouthparts, epipods, gills and gill formulae (Holthuis, 1993). We have tentatively selected four fossils corresponding to four deep nodes to calibrate our tree; three Upper Devonian and one Lower Triassic in age, as follows:

Node F1: *Aciculopoda mapesi* Feldmann & Schweitzer 2010 (Suborder Dendrobranchiata, Infraorder Penaeoidea)

This is the oldest fossil of a presumed dendrobranchiate shrimp known thus far. It was placed within the Penaeoidea, one of the two recognised dendrobranchiate infraorders (vs. Sergestoidea). The single specimen known was recovered from Famennian (374.5-359.2 MYPB), Late Devonian marine strata of Oklahoma (USA).

Node F2: *Palaeopalaemon newberryi* Whitfield, 1880 (Suborder Pleocyemata, Infraorder Palaeopalaemonida)

This is thus far the oldest decapod known, from Famennian (Late Devonian) marine deposits of Ohio, Kentucky, New York and Iowa (USA). Its precise taxonomic placement is controversial. Schram *et al.* (1978) placed *Palaeopalaemon* in the suborder Pleocyemata, suggesting affinities to glypheoids and astacideans. On the contrary, Felgenhauer & Abele (1983) disputed this assignment suggesting caridean or even dendrobranchiate kinship based on its shrimp-like antennal scaphocerite. Schram & Dixon (2004) considered *Palaeopalaemon* as sister to a clade comprising the Achelata, Anomala and Brachyura. Later on, De Grave *et al.* (2009) and Schweitzer *et al.* (2010) considered it to be a pleocyemate macruran and erected a new superfamily within the Astacidea to accommodate it. Meanwhile, Scholtz *et al.* (2009) suggested again its close affinity to the Dendrobranchiata. Most recently, Wahle *et al.* (2012) and Karasawa *et al.* (2013) recognized *Palaeopalaemon* as a member of the Reptantia but not as an astacidean, and erected a separate infraordinal status Palaeopalaemonida to accommodate it. Given the controversy over this issue, we have opted herein to consider *Palaeopalaemon* only as a stem pleocyemate.

Node F3: *Devonostenopus pennsylvaniensis* Jones, Feldmann, Schweitzer, Schram, Behr & Hand, 2014 (Suborder Pleocyemata, Infraorder Stenopodidea)

This fossil shrimp, recovered from presumed Late Devonian strata of Pennsylvania (USA), is considered to be the oldest known representative of the Stenepodidea. Even though its diagnosis as a stenopodidean is not entirely conclusive, the combination of features it shows suggests at least an affinity with this group rather than with caridean or dendrobranchiate decapods.

Node F4: *Upogebia obscura* (Von Meyer, 1834) (Suborder Pleocyemata, Infraorder Gebiidea)

This is presumably the oldest representative of the Gebiidea currently known, from the Lower Triassic of Germany. Data retrieved from Bracken-Grissom *et al.* (2014: table 1).

Tree calibration based on biogeographical events

As an alternative to fossil calibration, we explored the use of three younger biogeographical events assumed to have affected the diversification of particular TST shrimp lineages as described in Botello et al. (2013) as age constraint priors:

(1) The isolation of the populations of *T. galapagensis* from Santa Cruz and Isabela islands in the Galápagos, which is assumed not to be older than the age of the Cocos Ridge and associated seamounts. These now-submerged structures probably formed when the oceanic crust moved over the Galápagos hotspot, and it is probable that an archipelago has existed continuously above the current Galápagos area for the past 14.5 Ma (see Werner et al., 1999, and references therein), so the interval 5 – 14 Ma has been proposed for the separation of the two populations.

(2) The isolation of the ancestor of *Stygiocaris lancifera* and *S. stylifera* after the emergence of the Cape Range anticline in north-western Australia (7 – 10 Ma; Page et al., 2008).

(3) The occlusion of the Havana – Matanzas Channel in Cuba at 5 – 6 Ma (Iturralde-Vinent et al., 1996), which could have triggered the isolation of the ancestors of the sister species *T. consobrina* and *Typhlatya taina*.

Botello, A., Iliffe, T. M., Alvarez, F., Juan, C., Pons, J., & Jaume, D. 2013. Historical biogeography and phylogeny of *Typhlatya* cave shrimps (Decapoda: Atyidae) based on mitochondrial and nuclear data. *Journal of Biogeography* 40: 594–607.
Bracken-Grissom, H. D., Ahyong, S. T., Wilkinson, R. D., Feldmann, R. M., Schweitzer, C. E., Breinholt, J. W., Bendall, M., Palero, F., Chan, T.-Y., Felder, D. L., Robles, R., Chu, K.-H., Tsang, L.-M., Kim, D., Martin, J. W. & Crandall, K. A. 2014. The emergence of lobsters: Phylogenetic relationships, morphological evolution and divergence time comparisons of an ancient group (Decapoda: Achelata, Astacidea, Glypheidea, Polychelida). *Systematic Biology* 63: 457–479.
De Grave, S., N. D. Pentcheff, S. T. Ahyong, T.-Y. Chan, K. A. Crandall, P. C. Dworschak, D. L. Felder, R. M. Feldmann, C. H. I. M. Fransen, L. Y. D. Goulding, R. Lemaitre, M. L. Low, J. W. Martin, P. K. L. Ng, C. E. Schweitzer, S. H. Tan, D. Tshudy & R. Wetzer. 2009. A classification of Recent and fossil genera of decapod crustaceans. *The Raffles Bulletin of Zoology* Supplement 21:

1–109.
Feldmann, R.M. & Schweitzer, C.E. 2010. The oldest shrimp (Devonian: Famennian) and remarkable preservation of soft tissue. *Journal of Crustacean Biology*, 30: 629–635.
Felgenhauer, B. E. & L. G. Abele. 1983. Phylogenetic relationships among shrimp-like decapods. In: Schram, F. R. (ed.), *Crustacean Issues I, Crustacean Phylogeny*: 291–311. Rotterdam, Lisse: Balkema.
Holthuis, L. B. 1993. *The recent genera of the caridean and stenopodidean shrimps (Crustacea, Decapoda) with an appendix on the order Amphionidacea*. Nationaal Natuurhistorisch Museum, Leiden.
Iturralde-Vinent, M., Hubbell, G. & Rojas, R. 1996. Catalog of Cuban fossil Elasmobranchii (Paleocene–Pliocene) and paleogeographic implications of their Lower– Middle Miocene occurrence. *Boletín de la Sociedad Jamaicana de Geología* 31: 7–21.
Jones, W.T., Feldmann, R.M., Schweitzer, C.E., Schram, F.R., Behr, R. & Hand, K.L. 2014. The first Paleozoic stenopodidean from the Huntley Mountain Formation (Devonian-Carboniferous), north-central Pennsylvania. *Journal of Paleontology* 8: 1251–1256.
Karasawa, H., C. E. Schweitzer & R. M. Feldmann. 2013. Phylogeny and systematics of extant and extinct lobsters. *Journal of Crustacean Biology* 33: 78–123.
Page, T.J., Humphreys, W.F. & Hughes, J.M. 2008. Shrimps down under: evolutionary relationships of subterranean crustaceans from Western Australia (Decapoda: Atyidae: *Stygiocaris*). *PLoS ONE* 3: e1618.
Scholtz, G., A. Abzhanov, F. Alwes, C. Biffis & J. Pint. 2009. Development, genes and decapod evolution. In: Martin, J. W., K. A. Krandall & D. L. Felder (eds.), *Crustacean Issues 18, Decapod Crustacean Phylogenetics*: 31–46. CRC Press.
Schram, F. R. & C. J. Dixon. 2004. Decapod phylogeny: addition of fossil evidence to a robust morphological cladistic data set. *Bulletin of the Mizunami Fossil Museum* 31: 1–19.
Schram, F. R., R. M. Feldmann & M. J. Copeland. 1978. The Late Devonian Palaeopalaemonidae and the earliest decapod crustaceans. *Journal of Paleontology* 52: 1375–1387.
Schweitzer, C. E., R. M. Feldmann, A. Garassino, H. Karasawa & G. Schweigert. 2010. Systematic list of fossil decapod crustacean species. *Crustaceana Monographs* 10: 1–222.
Von Meyer, H. 1834. Krebse im bunten Sandstein. *Museum Senckenbergianum*, 1: 293–295.
Wahle, R. A., D. Tshudy, J. S. Cobb, J. Factor & M. Jaini. 2012. Infraorder Astacidea Latreille, 1802 P.P.: The Marine Clawed Lobsters. In: F. R. Schram, J. C. von Vaupel Klein, M. Charmantier-Daures & J. Forest (eds.), *Treatise on Zoology – Anatomy, Taxonomy, Biology. The Crustacea. Vol. 9, Part B,* pp. 3–108. *Eucarida: Decapoda: Astacidea P.P. (Enoplometopoidea, Nephropoidea), Glypheidea, Axiidea, Gebiidea, and Anomura*. Brill, Leiden and Boston.
Werner, R., Hoernle, K., van den Bogaard, P., Ranero, C., von Huene, R. & Korich, D. 1999. Drowned 14-m.y.-old Galápagos archipelago off the coast of Costa Rica: implications for tectonic and evolutionary models. *Geology* 27: 400–502.
Whitfield, R. P. 1880. Notice of new forms of fossil crustaceans from the Upper Devonian rocks of Ohio, with descriptions of new genera and species. *American Journal of Science* 3: 33–42.

Supplementary Figure 1. Phylogenetic trees obtained in the partitioning analyses under a maximum likelihood (ML) framework using IQTREE (a-m) and under the Bayesian mixture CAT model in PHYLOBAYES (n and o). The resulting tree derived from the exclusion of the third coding positions in the alignment (p) and the tree obtained using the *cox*1 gene only (q) are also shown. Numbers at the right of each node represent bootstrap (ML trees) or Bayesian probability (PHYLOBAYES trees) supports. The partitioning scheme and the evolutionary models assigned to each subset are also shown. Figure starts in the next page.

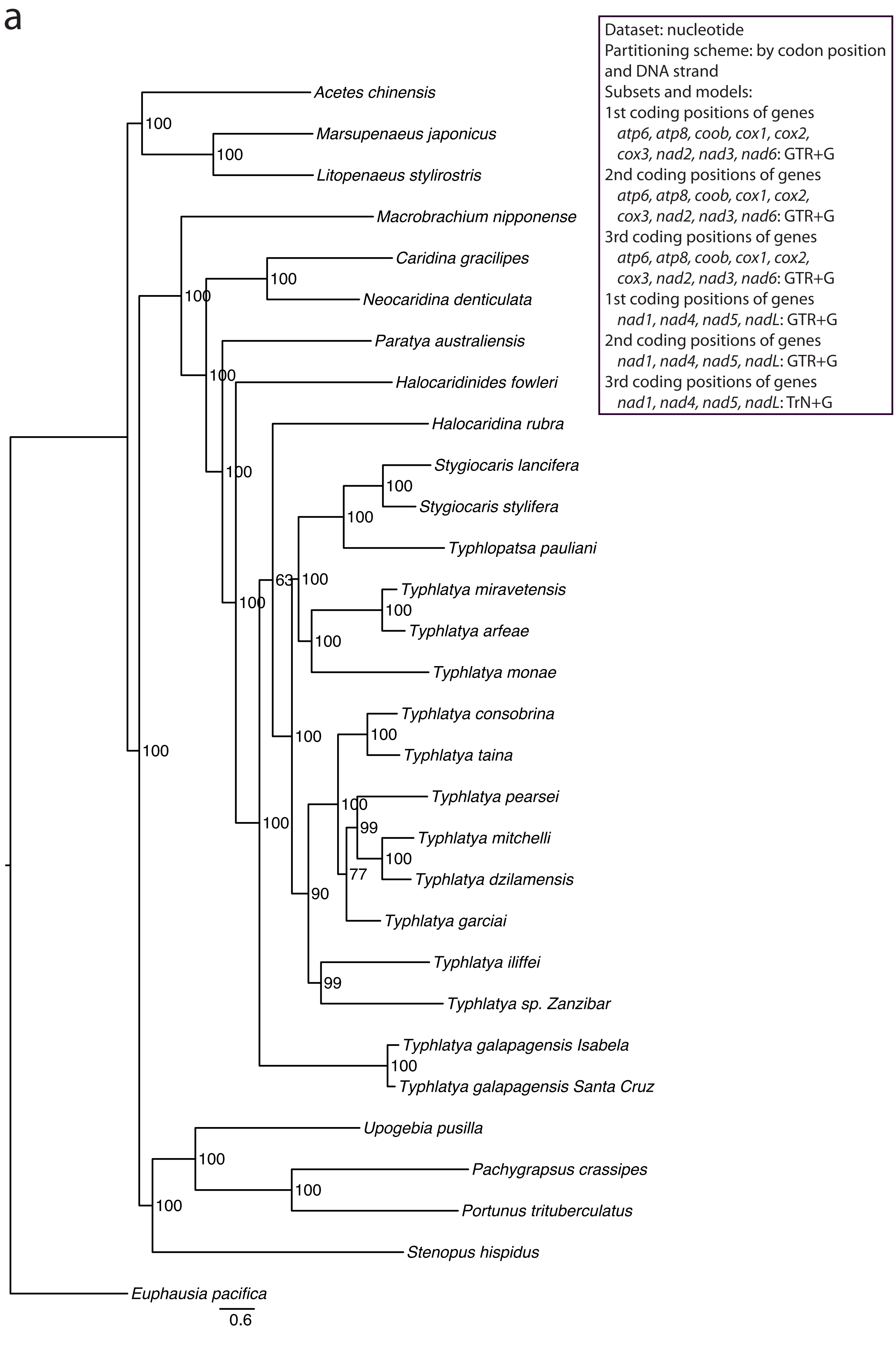
a
Dataset: nucleotide
Partitioning scheme: by codon position and DNA strand
Subsets and models:
1st coding positions of genes atp6, atp8, coob, cox1, cox2, cox3, nad2, nad3, nad6: GTR+G
2nd coding positions of genes atp6, atp8, coob, cox1, cox2, cox3, nad2, nad3, nad6: GTR+G
3rd coding positions of genes atp6, atp8, coob, cox1, cox2, cox3, nad2, nad3, nad6: GTR+G
1st coding positions of genes nad1, nad4, nad5, nadL: GTR+G
2nd coding positions of genes nad1, nad4, nad5, nadL: GTR+G
3rd coding positions of genes nad1, nad4, nad5, nadL: TrN+G
Acetes chinensis
Marsupenaeus japonicus
Litopenaeus stylirostris
Macrobrachium nipponense
Caridina gracilipes
Neocaridina denticulata
Paratya australiensis
Halocaridinides fowleri
Halocaridina rubra
Stygiocaris lancifera
Stygiocaris stylifera
Typhlopatsa pauliani
Typhlatya miravetensis
Typhlatya arfeae
Typhlatya monae
Typhlatya consobrina
Typhlatya taina
Typhlatya pearsei
Typhlatya mitchelli
Typhlatya dzilamensis
Typhlatya garciai
Typhlatya iliffei
Typhlatya sp. Zanzibar
Typhlatya galapagensis Isabela
Typhlatya galapagensis Santa Cruz
Upogebia pusilla
Pachygrapsus crassipes
Portunus trituberculatus
Stenopus hispidus
Euphausia pacifica
100
63
99
77
90
0.6

b

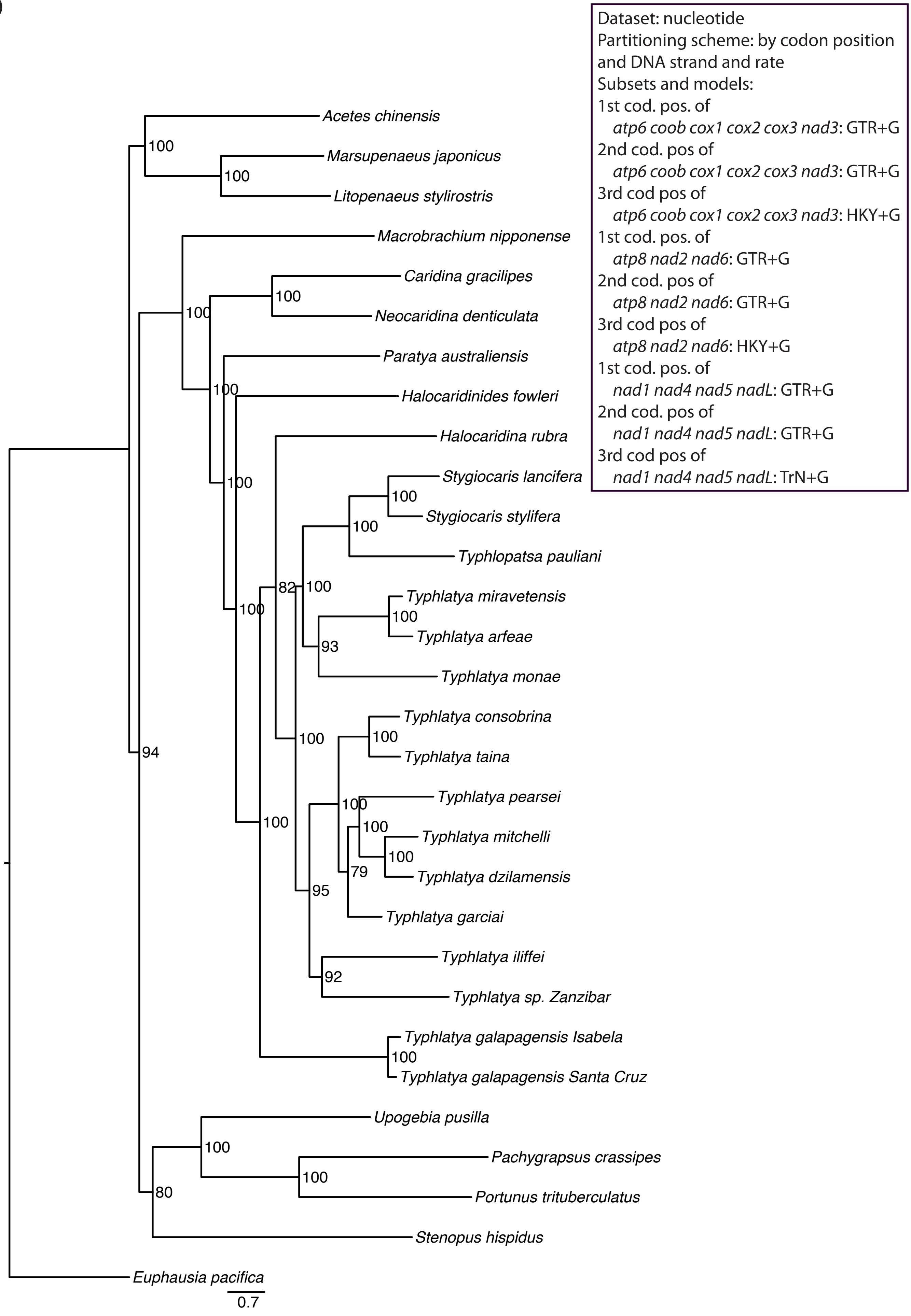
Dataset: nucleotide
Partitioning scheme: by codon position and DNA strand and rate
Subsets and models:
1st cod. pos. of
atp6 coob cox1 cox2 cox3 nad3: GTR+G
2nd cod. pos of
atp6 coob cox1 cox2 cox3 nad3: GTR+G
3rd cod pos of
atp6 coob cox1 cox2 cox3 nad3: HKY+G
1st cod. pos. of
atp8 nad2 nad6: GTR+G
2nd cod. pos of
atp8 nad2 nad6: GTR+G
3rd cod pos of
atp8 nad2 nad6: HKY+G
1st cod. pos. of
nad1 nad4 nad5 nadL: GTR+G
2nd cod. pos of
nad1 nad4 nad5 nadL: GTR+G
3rd cod pos of
nad1 nad4 nad5 nadL: TrN+G
Acetes chinensis
Marsupenaeus japonicus
Litopenaeus stylirostris
Macrobrachium nipponense
Caridina gracilipes
Neocaridina denticulata
Paratya australiensis
Halocaridinides fowleri
Halocaridina rubra
Stygiocaris lancifera
Stygiocaris stylifera
Typhlopatsa pauliani
Typhlatya miravetensis
Typhlatya arfeae
Typhlatya monae
Typhlatya consobrina
Typhlatya taina
Typhlatya pearsei
Typhlatya mitchelli
Typhlatya dzilamensis
Typhlatya garciai
Typhlatya iliffei
Typhlatya sp. Zanzibar
Typhlatya galapagensis Isabela
Typhlatya galapagensis Santa Cruz
Upogebia pusilla
Pachygrapsus crassipes
Portunus trituberculatus
Stenopus hispidus
Euphausia pacifica
0.7

C

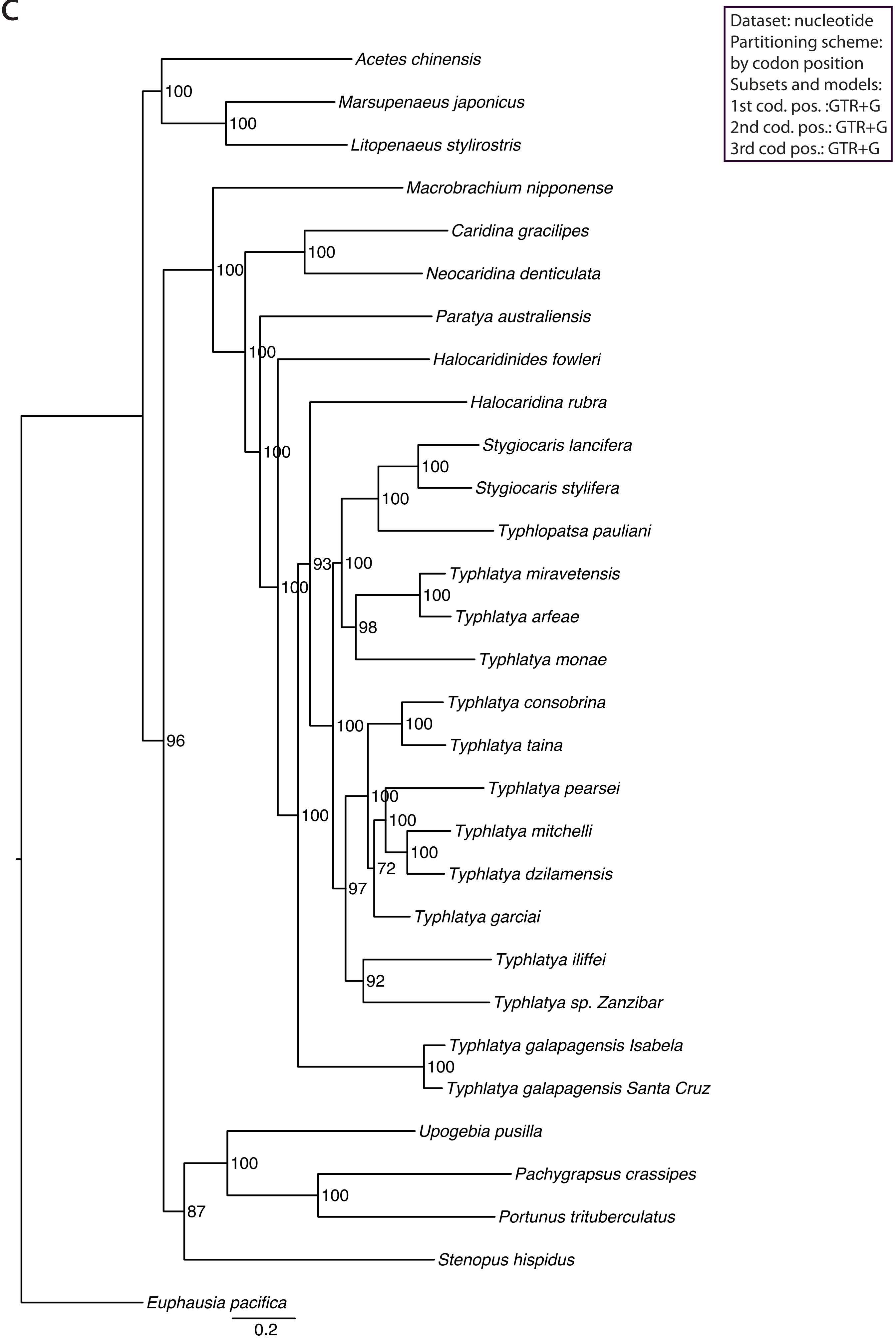
Dataset: nucleotide
Partitioning scheme:
by codon position
Subsets and models:
1st cod. pos. :GTR+G
2nd cod. pos.: GTR+G
3rd cod pos.: GTR+G
Acetes chinensis
Marsupenaeus japonicus
Litopenaeus stylirostris
Macrobrachium nipponense
Caridina gracilipes
Neocaridina denticulata
Paratya australiensis
Halocaridinides fowleri
Halocaridina rubra
Stygiocaris lancifera
Stygiocaris stylifera
Typhlopatsa pauliani
Typhlatya miravetensis
Typhlatya arfeae
Typhlatya monae
Typhlatya consobrina
Typhlatya taina
Typhlatya pearsei
Typhlatya mitchelli
Typhlatya dzilamensis
Typhlatya garciai
Typhlatya iliffei
Typhlatya sp. Zanzibar
Typhlatya galapagensis Isabela
Typhlatya galapagensis Santa Cruz
Upogebia pusilla
Pachygrapsus crassipes
Portunus trituberculatus
Stenopus hispidus
Euphausia pacifica
100
100
100
100
100
100
100
100
100
93
100
100
98
100
100
100
100
100
72
97
92
100
96
100
100
87
0.2

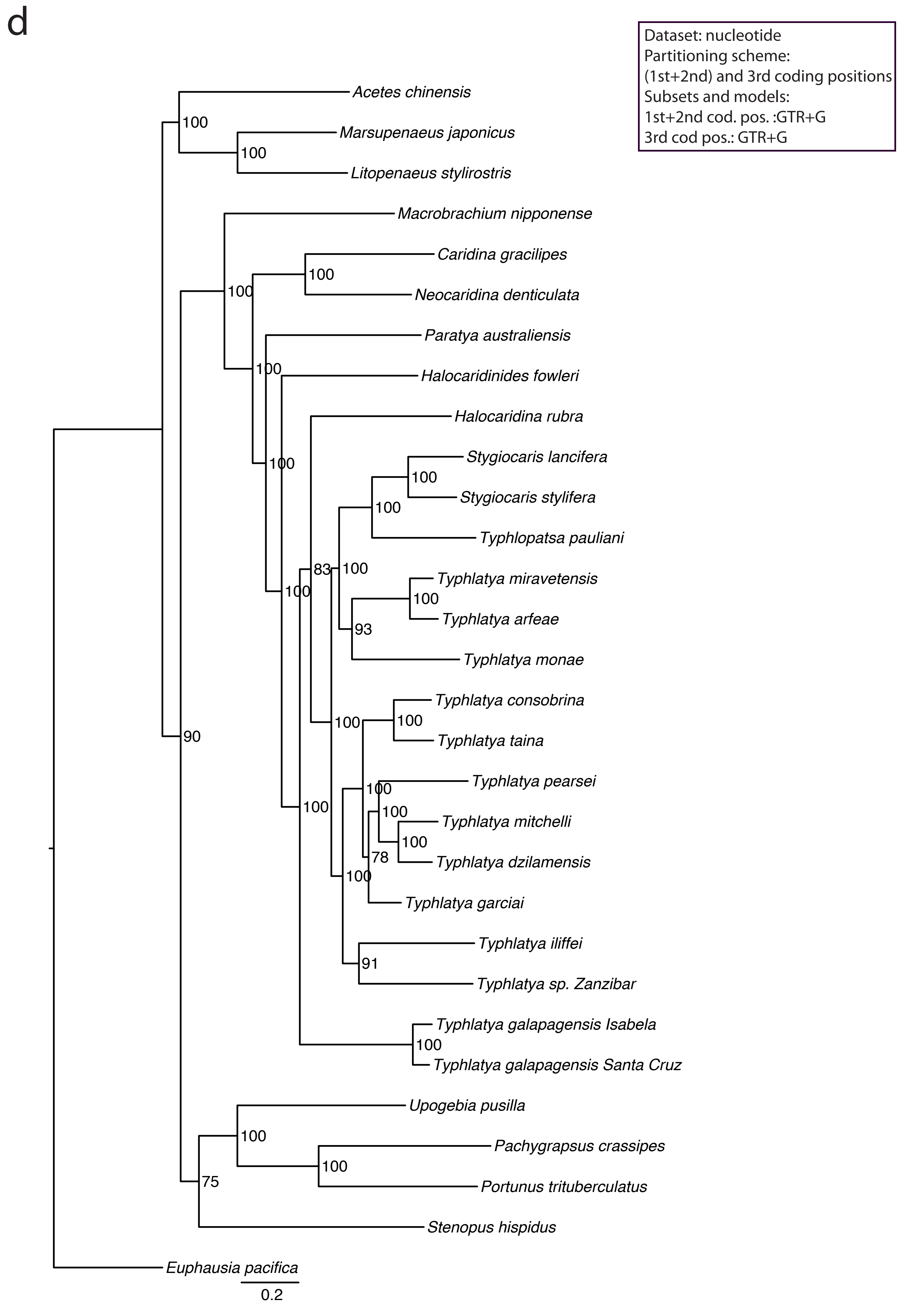
d
Dataset: nucleotide
Partitioning scheme:
(1st+2nd) and 3rd coding positions
Subsets and models:
1st+2nd cod. pos. :GTR+G
3rd cod pos.: GTR+G
Acetes chinensis
Marsupenaeus japonicus
Litopenaeus stylirostris
Macrobrachium nipponense
Caridina gracilipes
Neocaridina denticulata
Paratya australiensis
Halocaridinides fowleri
Halocaridina rubra
Stygiocaris lancifera
Stygiocaris stylifera
Typhlopatsa pauliani
Typhlatya miravetensis
Typhlatya arfeae
Typhlatya monae
Typhlatya consobrina
Typhlatya taina
Typhlatya pearsei
Typhlatya mitchelli
Typhlatya dzilamensis
Typhlatya garciai
Typhlatya iliffei
Typhlatya sp. Zanzibar
Typhlatya galapagensis Isabela
Typhlatya galapagensis Santa Cruz
Upogebia pusilla
Pachygrapsus crassipes
Portunus trituberculatus
Stenopus hispidus
Euphausia pacifica
0.2

e

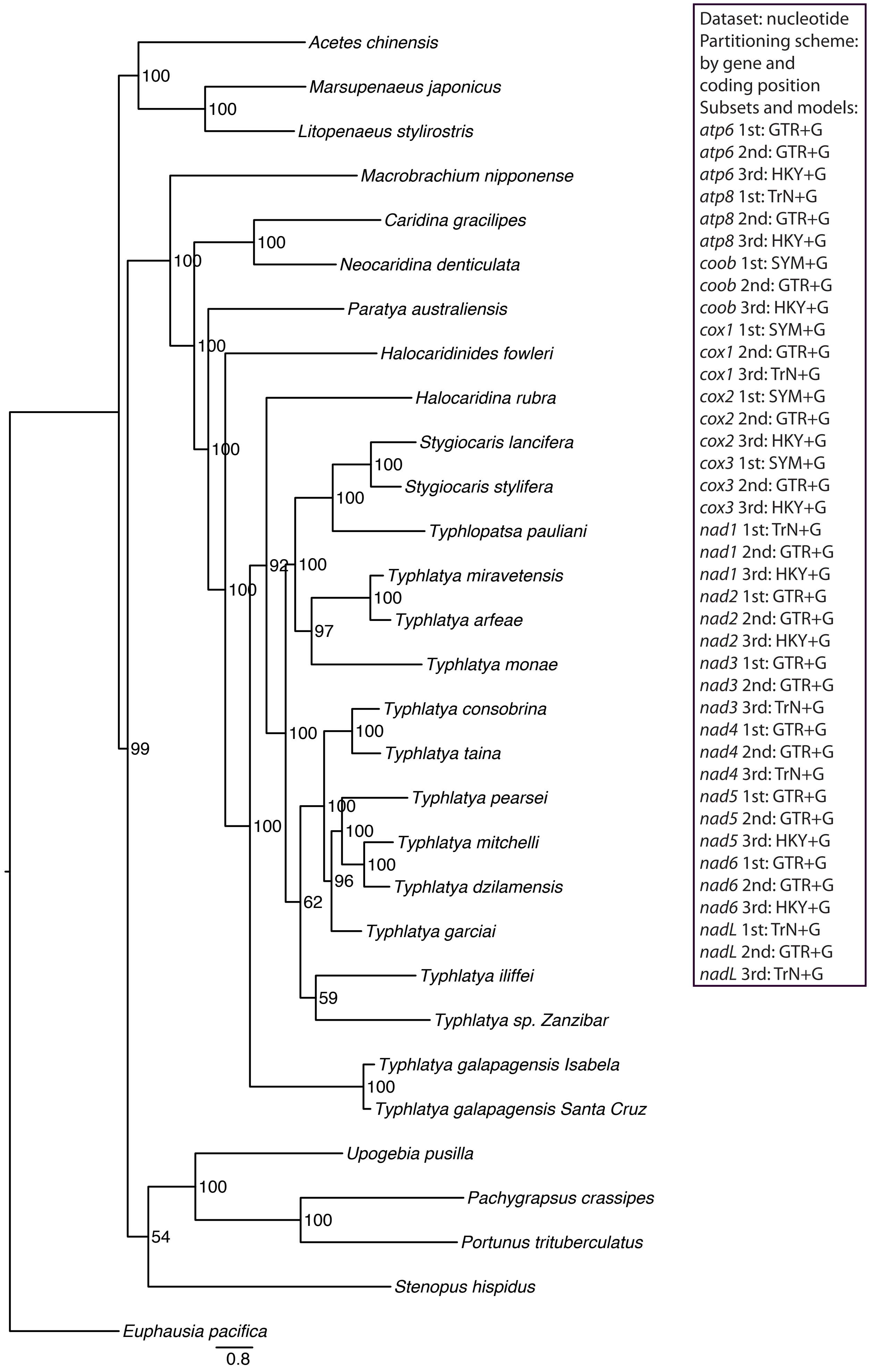
Acetes chinensis
Marsupenaeus japonicus
Litopenaeus stylirostris
Macrobrachium nipponense
Caridina gracilipes
Neocaridina denticulata
Paratya australiensis
Halocaridinides fowleri
Halocaridina rubra
Stygiocaris lancifera
Stygiocaris stylifera
Typhlopatsa pauliani
Typhlatya miravetensis
Typhlatya arfeae
Typhlatya monae
Typhlatya consobrina
Typhlatya taina
Typhlatya pearsei
Typhlatya mitchelli
Typhlatya dzilamensis
Typhlatya garciai
Typhlatya iliffei
Typhlatya sp. Zanzibar
Typhlatya galapagensis Isabela
Typhlatya galapagensis Santa Cruz
Upogebia pusilla
Pachygrapsus crassipes
Portunus trituberculatus
Stenopus hispidus
Euphausia pacifica
0.8
Dataset: nucleotide
Partitioning scheme: by gene and coding position
Subsets and models:
atp6 1st: GTR+G
atp6 2nd: GTR+G
atp6 3rd: HKY+G
atp8 1st: TrN+G
atp8 2nd: GTR+G
atp8 3rd: HKY+G
coob 1st: SYM+G
coob 2nd: GTR+G
coob 3rd: HKY+G
cox1 1st: SYM+G
cox1 2nd: GTR+G
cox1 3rd: TrN+G
cox2 1st: SYM+G
cox2 2nd: GTR+G
cox2 3rd: HKY+G
cox3 1st: SYM+G
cox3 2nd: GTR+G
cox3 3rd: HKY+G
nad1 1st: TrN+G
nad1 2nd: GTR+G
nad1 3rd: HKY+G
nad2 1st: GTR+G
nad2 2nd: GTR+G
nad2 3rd: HKY+G
nad3 1st: GTR+G
nad3 2nd: GTR+G
nad3 3rd: TrN+G
nad4 1st: GTR+G
nad4 2nd: GTR+G
nad4 3rd: TrN+G
nad5 1st: GTR+G
nad5 2nd: GTR+G
nad5 3rd: HKY+G
nad6 1st: GTR+G
nad6 2nd: GTR+G
nad6 3rd: HKY+G
nadL 1st: TrN+G
nadL 2nd: GTR+G
nadL 3rd: TrN+G

f

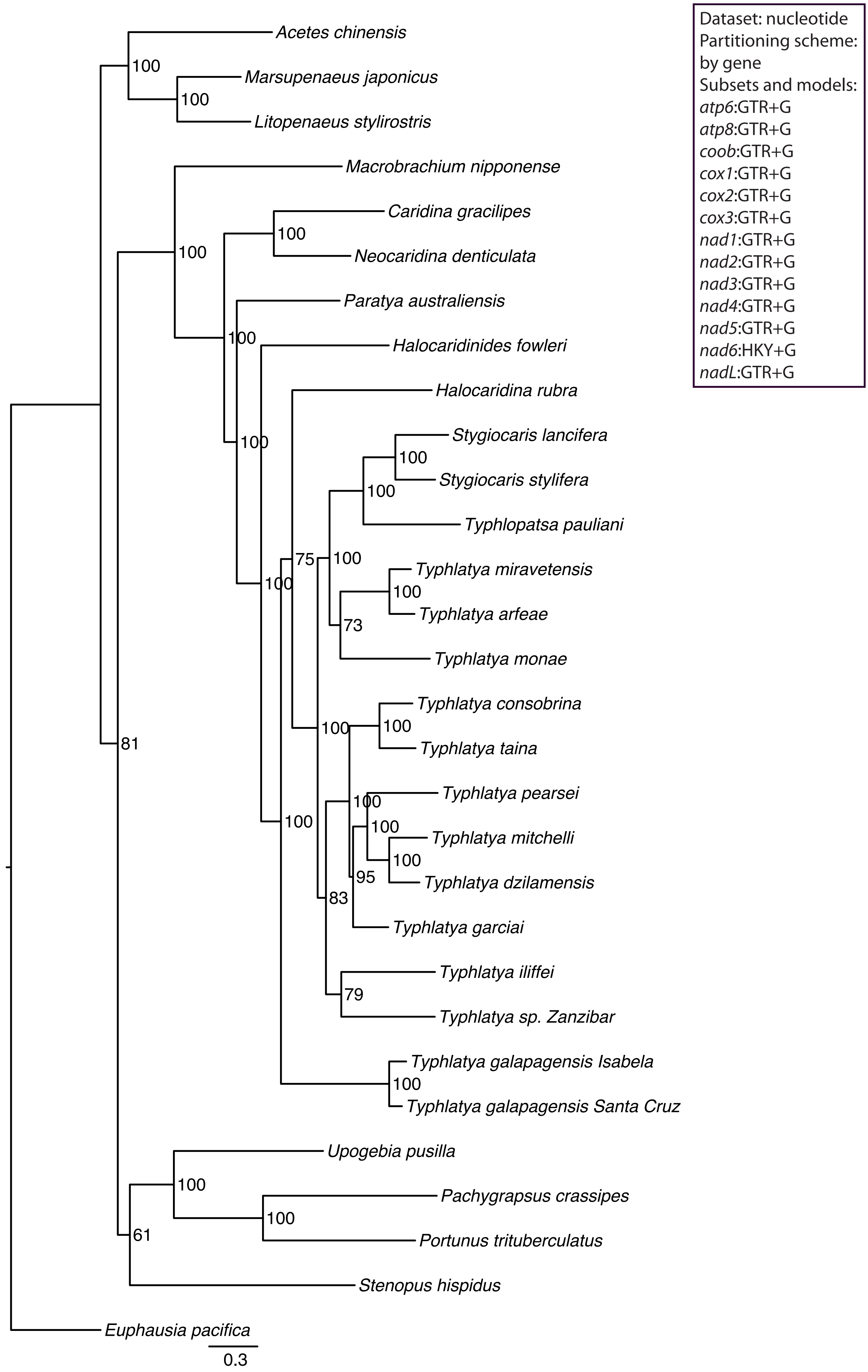
Acetes chinensis
Marsupenaeus japonicus
Litopenaeus stylirostris
Macrobrachium nipponense
Caridina gracilipes
Neocaridina denticulata
Paratya australiensis
Halocaridinides fowleri
Halocaridina rubra
Stygiocaris lancifera
Stygiocaris stylifera
Typhlopatsa pauliani
Typhlatya miravetensis
Typhlatya arfeae
Typhlatya monae
Typhlatya consobrina
Typhlatya taina
Typhlatya pearsei
Typhlatya mitchelli
Typhlatya dzilamensis
Typhlatya garciai
Typhlatya iliffei
Typhlatya sp. Zanzibar
Typhlatya galapagensis Isabela
Typhlatya galapagensis Santa Cruz
Upogebia pusilla
Pachygrapsus crassipes
Portunus trituberculatus
Stenopus hispidus
Euphausia pacifica
100
100
100
100
100
100
100
100
100
75
100
100
73
100
100
100
100
100
100
95
83
79
100
81
100
100
61
0.3
Dataset: nucleotide
Partitioning scheme: by gene
Subsets and models:
atp6:GTR+G
atp8:GTR+G
coob:GTR+G
cox1:GTR+G
cox2:GTR+G
cox3:GTR+G
nad1:GTR+G
nad2:GTR+G
nad3:GTR+G
nad4:GTR+G
nad5:GTR+G
nad6:HKY+G
nadL:GTR+G

g

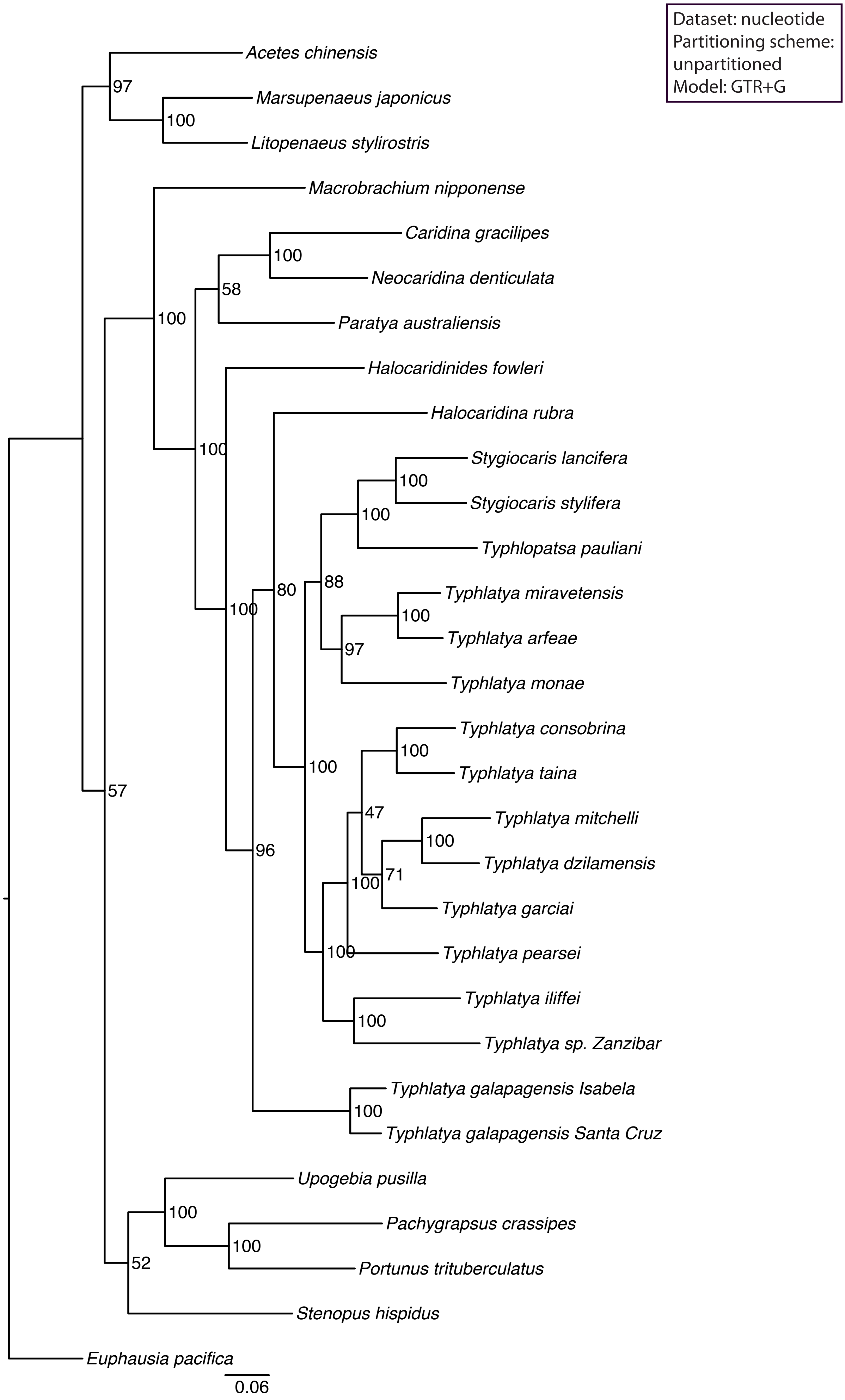
Dataset: nucleotide
Partitioning scheme: unpartitioned
Model: GTR+G
Acetes chinensis
97
Marsupenaeus japonicus
100
Litopenaeus stylirostris
Macrobrachium nipponense
Caridina gracilipes
100
Neocaridina denticulata
58
100
Paratya australiensis
Halocaridinides fowleri
Halocaridina rubra
100
Stygiocaris lancifera
100
Stygiocaris stylifera
100
Typhlopatsa pauliani
80
88
Typhlatya miravetensis
100
100
Typhlatya arfeae
97
Typhlatya monae
Typhlatya consobrina
100
Typhlatya taina
100
57
47
Typhlatya mitchelli
100
Typhlatya dzilamensis
71
100
Typhlatya garciai
96
100
Typhlatya pearsei
Typhlatya iliffei
100
Typhlatya sp. Zanzibar
Typhlatya galapagensis Isabela
100
Typhlatya galapagensis Santa Cruz
Upogebia pusilla
100
Pachygrapsus crassipes
100
Portunus trituberculatus
52
Stenopus hispidus
Euphausia pacifica
0.06

h

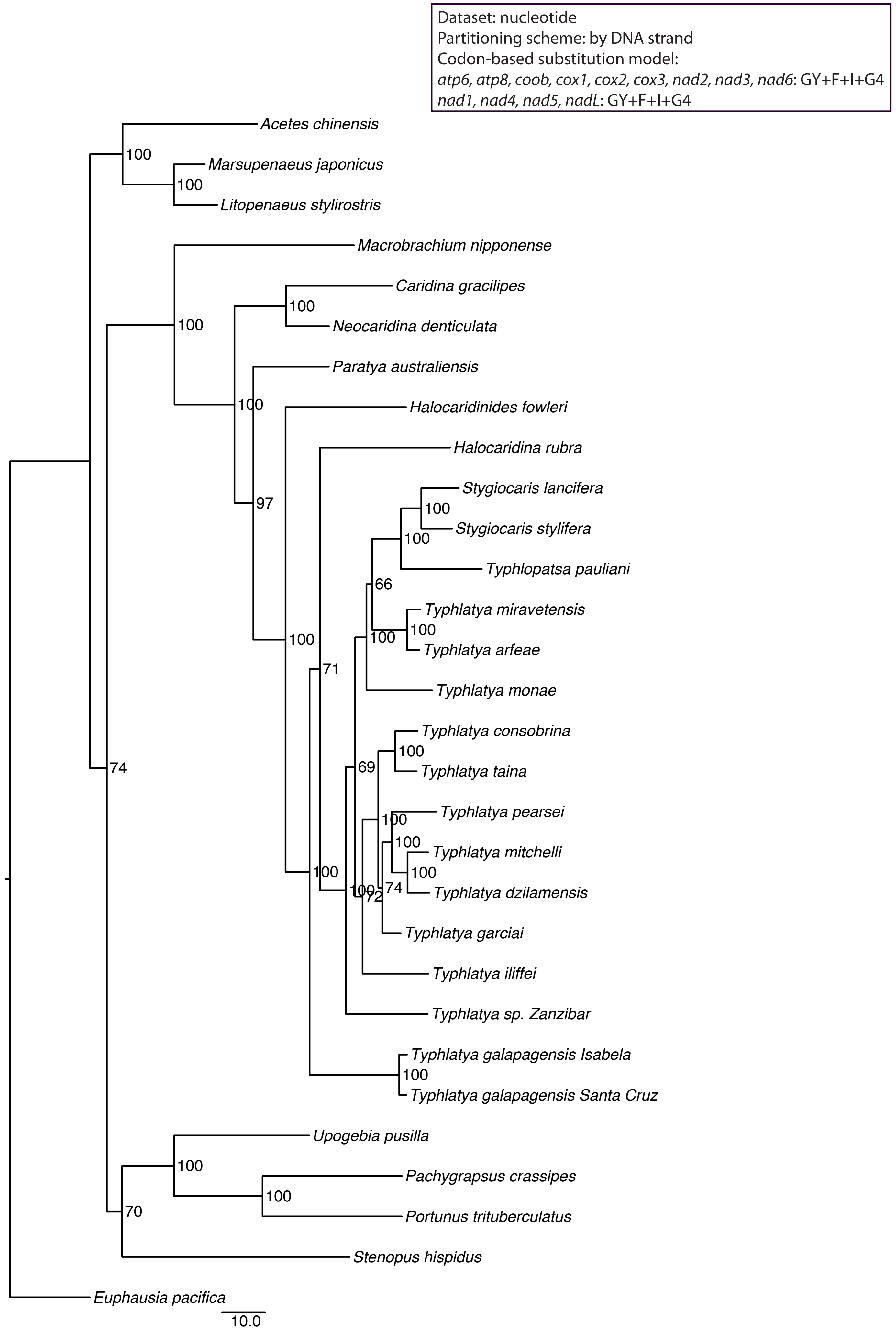
Dataset: nucleotide
Partitioning scheme: by DNA strand
Codon-based substitution model:
atp6, atp8, coob, cox1, cox2, cox3, nad2, nad3, nad6: GY+F+I+G4
nad1, nad4, nad5, nadL: GY+F+I+G4
Acetes chinensis
Marsupenaeus japonicus
Litopenaeus stylirostris
Macrobrachium nipponense
Caridina gracilipes
Neocaridina denticulata
Paratya australiensis
Halocaridinides fowleri
Halocaridina rubra
Stygiocaris lancifera
Stygiocaris stylifera
Typhlopatsa pauliani
Typhlatya miravetensis
Typhlatya arfeae
Typhlatya monae
Typhlatya consobrina
Typhlatya taina
Typhlatya pearsei
Typhlatya mitchelli
Typhlatya dzilamensis
Typhlatya garciai
Typhlatya iliffei
Typhlatya sp. Zanzibar
Typhlatya galapagensis Isabela
Typhlatya galapagensis Santa Cruz
Upogebia pusilla
Pachygrapsus crassipes
Portunus trituberculatus
Stenopus hispidus
Euphausia pacifica
100
100
100
100
100
97
100
71
100
100
66
100
100
100
69
100
100
100
74
72
100
100
74
100
100
70
10.0

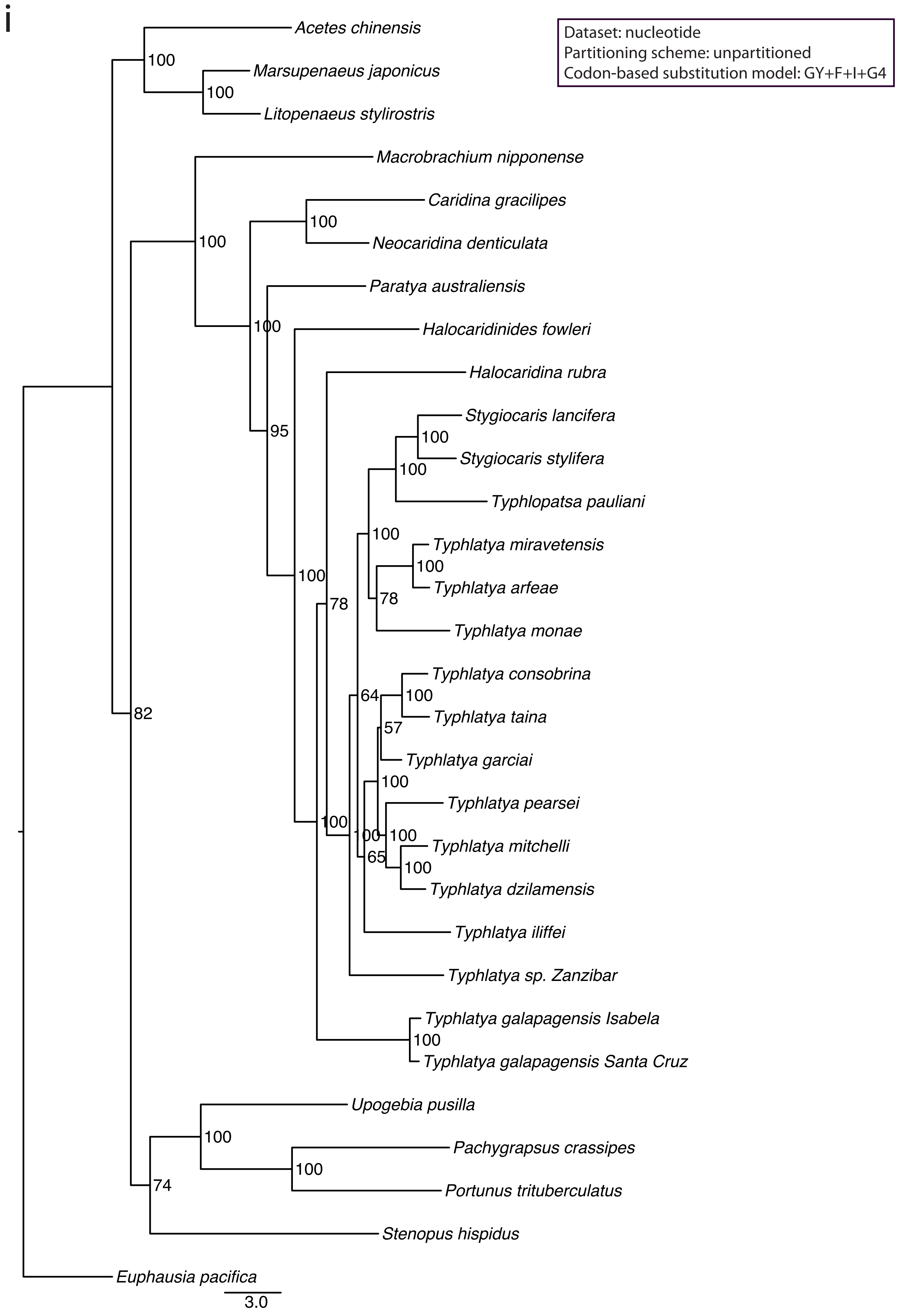

i
Dataset: nucleotide
Partitioning scheme: unpartitioned
Codon-based substitution model: GY+F+I+G4
Acetes chinensis
Marsupenaeus japonicus
Litopenaeus stylirostris
Macrobrachium nipponense
Caridina gracilipes
Neocaridina denticulata
Paratya australiensis
Halocaridinides fowleri
Halocaridina rubra
Stygiocaris lancifera
Stygiocaris stylifera
Typhlopatsa pauliani
Typhlatya miravetensis
Typhlatya arfeae
Typhlatya monae
Typhlatya consobrina
Typhlatya taina
Typhlatya garciai
Typhlatya pearsei
Typhlatya mitchelli
Typhlatya dzilamensis
Typhlatya iliffei
Typhlatya sp. Zanzibar
Typhlatya galapagensis Isabela
Typhlatya galapagensis Santa Cruz
Upogebia pusilla
Pachygrapsus crassipes
Portunus trituberculatus
Stenopus hispidus
Euphausia pacifica
3.0

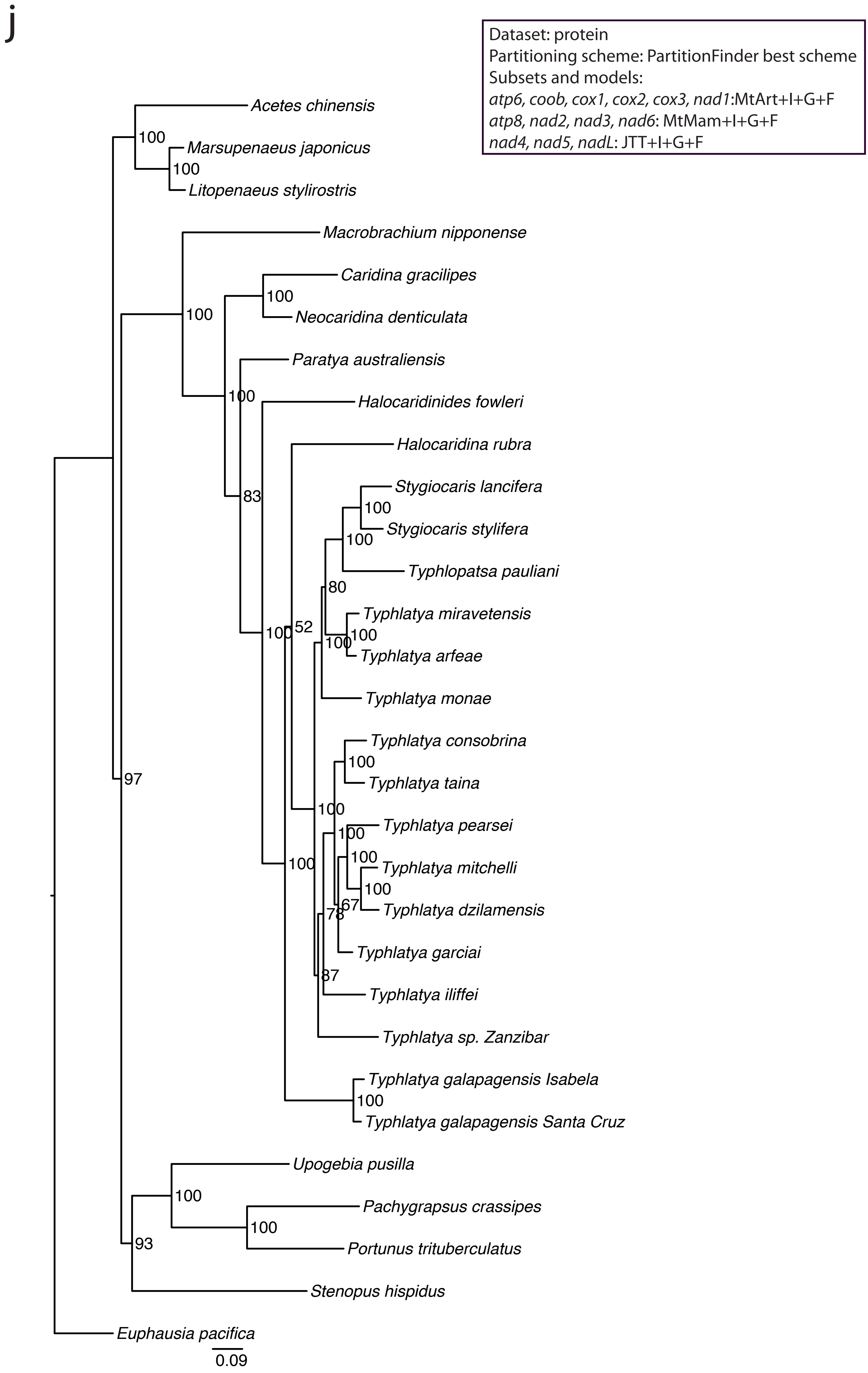
j
Dataset: protein
Partitioning scheme: PartitionFinder best scheme
Subsets and models:
atp6, coob, cox1, cox2, cox3, nad1:MtArt+I+G+F
atp8, nad2, nad3, nad6: MtMam+I+G+F
nad4, nad5, nadL: JTT+I+G+F
Acetes chinensis
Marsupenaeus japonicus
Litopenaeus stylirostris
Macrobrachium nipponense
Caridina gracilipes
Neocaridina denticulata
Paratya australiensis
Halocaridinides fowleri
Halocaridina rubra
Stygiocaris lancifera
Stygiocaris stylifera
Typhlopatsa pauliani
Typhlatya miravetensis
Typhlatya arfeae
Typhlatya monae
Typhlatya consobrina
Typhlatya taina
Typhlatya pearsei
Typhlatya mitchelli
Typhlatya dzilamensis
Typhlatya garciai
Typhlatya iliffei
Typhlatya sp. Zanzibar
Typhlatya galapagensis Isabela
Typhlatya galapagensis Santa Cruz
Upogebia pusilla
Pachygrapsus crassipes
Portunus trituberculatus
Stenopus hispidus
Euphausia pacifica
100
100
100
100
100
100
83
80
100
100
100
100
52
100
100
100
100
100
100
100
67
78
87
100
97
100
100
93
0.09

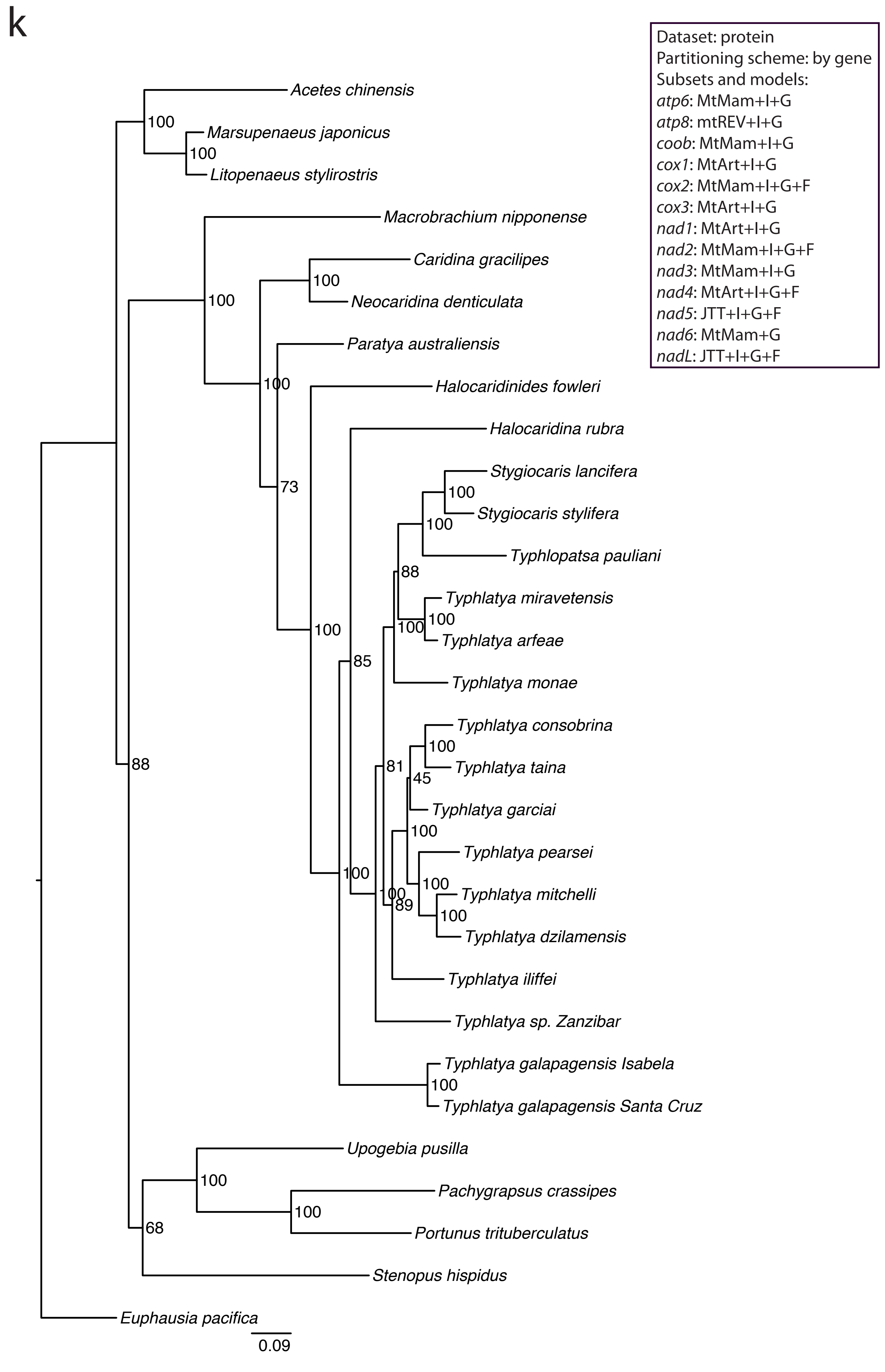
k
Dataset: protein
Partitioning scheme: by gene
Subsets and models:
atp6: MtMam+I+G
atp8: mtREV+I+G
coob: MtMam+I+G
cox1: MtArt+I+G
cox2: MtMam+I+G+F
cox3: MtArt+I+G
nad1: MtArt+I+G
nad2: MtMam+I+G+F
nad3: MtMam+I+G
nad4: MtArt+I+G+F
nad5: JTT+I+G+F
nad6: MtMam+G
nadL: JTT+I+G+F
Acetes chinensis
Marsupenaeus japonicus
Litopenaeus stylirostris
Macrobrachium nipponense
Caridina gracilipes
Neocaridina denticulata
Paratya australiensis
Halocaridinides fowleri
Halocaridina rubra
Stygiocaris lancifera
Stygiocaris stylifera
Typhlopatsa pauliani
Typhlatya miravetensis
Typhlatya arfeae
Typhlatya monae
Typhlatya consobrina
Typhlatya taina
Typhlatya garciai
Typhlatya pearsei
Typhlatya mitchelli
Typhlatya dzilamensis
Typhlatya iliffei
Typhlatya sp. Zanzibar
Typhlatya galapagensis Isabela
Typhlatya galapagensis Santa Cruz
Upogebia pusilla
Pachygrapsus crassipes
Portunus trituberculatus
Stenopus hispidus
Euphausia pacifica
0.09

I

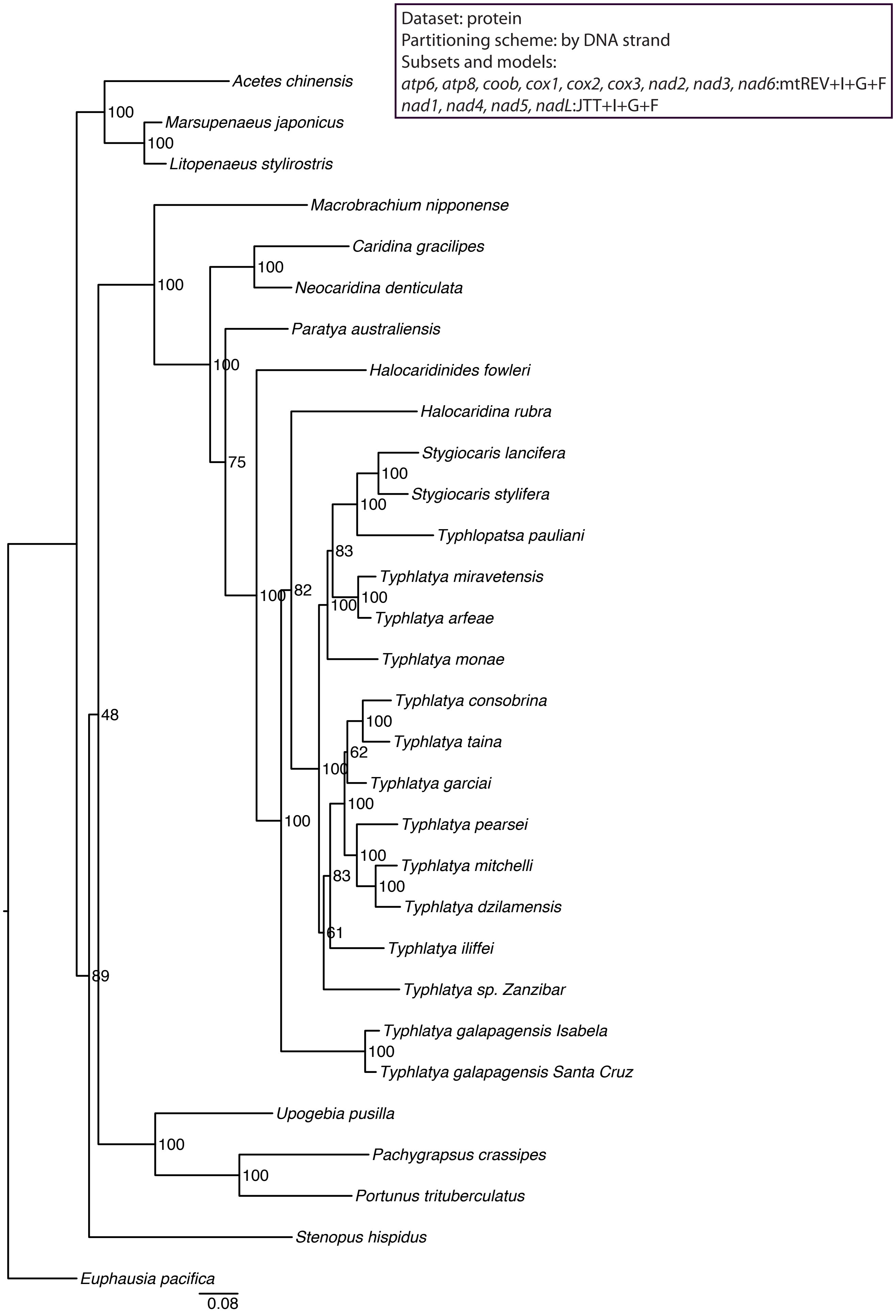
Dataset: protein
Partitioning scheme: by DNA strand
Subsets and models:
atp6, atp8, coob, cox1, cox2, cox3, nad2, nad3, nad6:mtREV+I+G+F
nad1, nad4, nad5, nadL:JTT+I+G+F
Acetes chinensis
Marsupenaeus japonicus
Litopenaeus stylirostris
Macrobrachium nipponense
Caridina gracilipes
Neocaridina denticulata
Paratya australiensis
Halocaridinides fowleri
Halocaridina rubra
Stygiocaris lancifera
Stygiocaris stylifera
Typhlopatsa pauliani
Typhlatya miravetensis
Typhlatya arfeae
Typhlatya monae
Typhlatya consobrina
Typhlatya taina
Typhlatya garciai
Typhlatya pearsei
Typhlatya mitchelli
Typhlatya dzilamensis
Typhlatya iliffei
Typhlatya sp. Zanzibar
Typhlatya galapagensis Isabela
Typhlatya galapagensis Santa Cruz
Upogebia pusilla
Pachygrapsus crassipes
Portunus trituberculatus
Stenopus hispidus
Euphausia pacifica
100
100
100
100
100
75
100
82
100
83
100
100
100
48
100
62
100
100
83
100
61
100
89
100
100
100
0.08

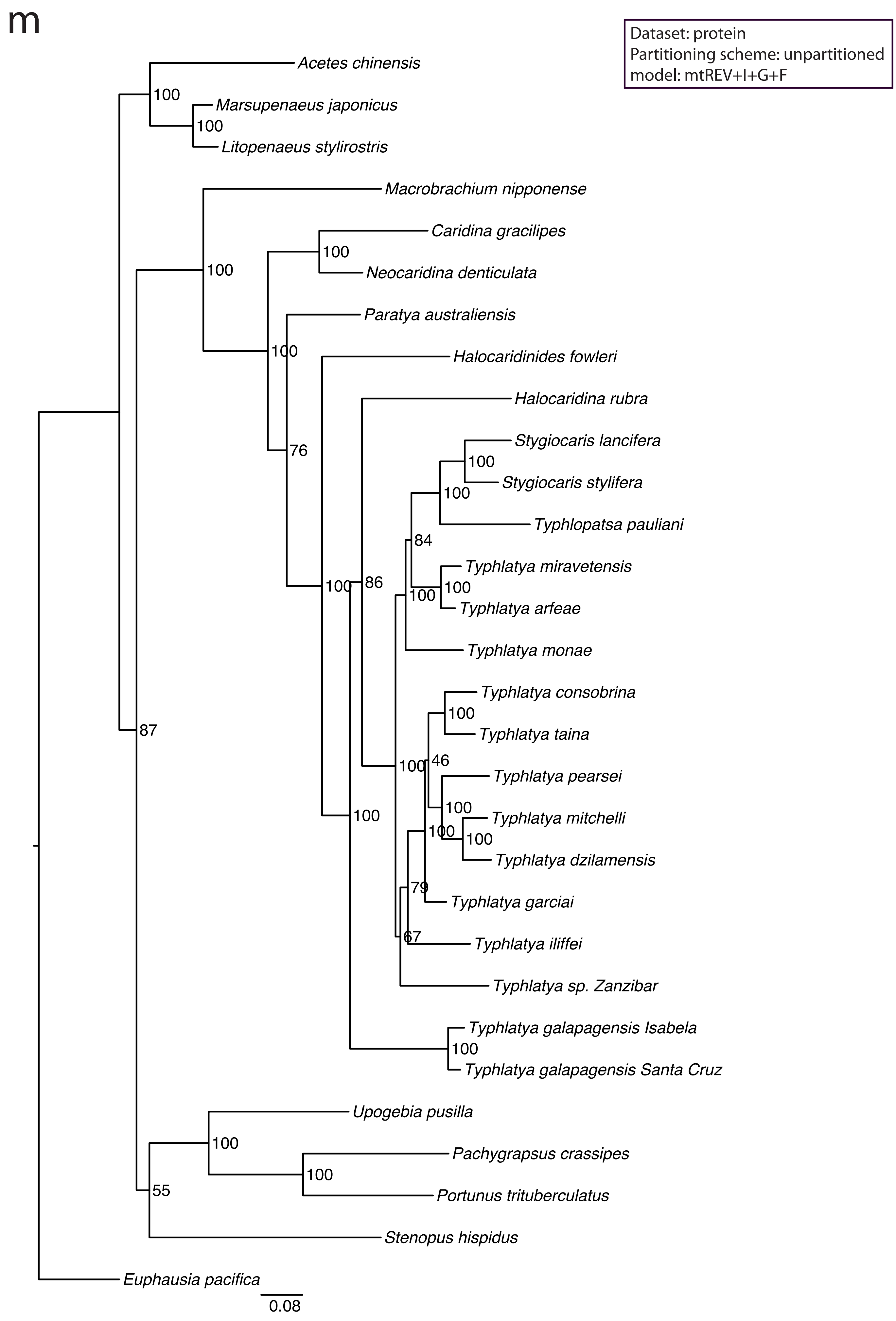
m
Dataset: protein
Partitioning scheme: unpartitioned
model: mtREV+I+G+F
Acetes chinensis
Marsupenaeus japonicus
Litopenaeus stylirostris
Macrobrachium nipponense
Caridina gracilipes
Neocaridina denticulata
Paratya australiensis
Halocaridinides fowleri
Halocaridina rubra
Stygiocaris lancifera
Stygiocaris stylifera
Typhlopatsa pauliani
Typhlatya miravetensis
Typhlatya arfeae
Typhlatya monae
Typhlatya consobrina
Typhlatya taina
Typhlatya pearsei
Typhlatya mitchelli
Typhlatya dzilamensis
Typhlatya garciai
Typhlatya iliffei
Typhlatya sp. Zanzibar
Typhlatya galapagensis Isabela
Typhlatya galapagensis Santa Cruz
Upogebia pusilla
Pachygrapsus crassipes
Portunus trituberculatus
Stenopus hispidus
Euphausia pacifica
100
100
100
100
100
76
100
86
100
100
84
100
100
100
46
100
100
100
100
79
67
100
87
100
100
55
0.08

n

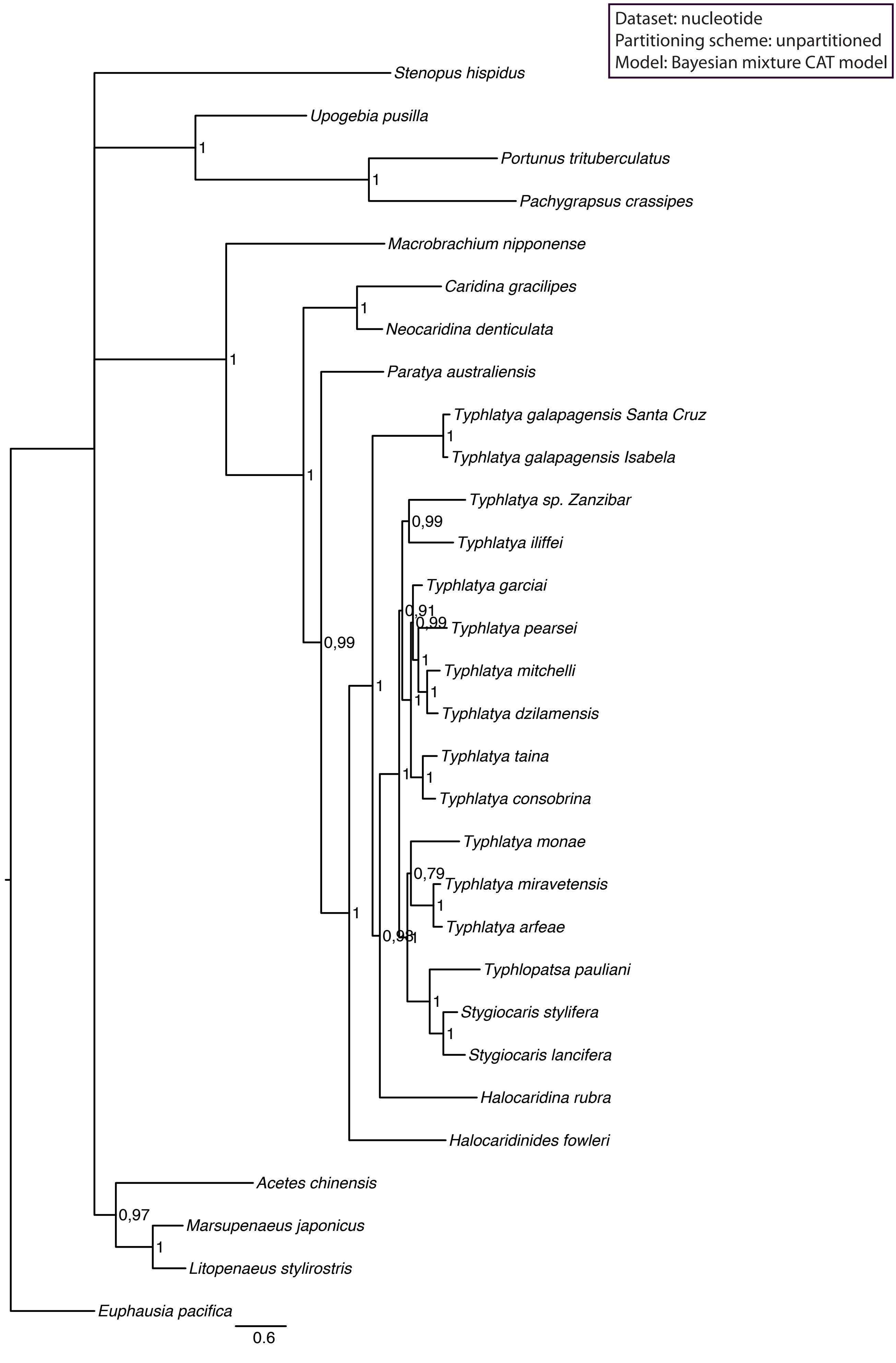

Dataset: nucleotide
Partitioning scheme: unpartitioned
Model: Bayesian mixture CAT model
Stenopus hispidus
Upogebia pusilla
Portunus trituberculatus
Pachygrapsus crassipes
Macrobrachium nipponense
Caridina gracilipes
Neocaridina denticulata
Paratya australiensis
Typhlatya galapagensis Santa Cruz
Typhlatya galapagensis Isabela
Typhlatya sp. Zanzibar
Typhlatya iliffei
Typhlatya garciai
Typhlatya pearsei
Typhlatya mitchelli
Typhlatya dzilamensis
Typhlatya taina
Typhlatya consobrina
Typhlatya monae
Typhlatya miravetensis
Typhlatya arfeae
Typhlopatsa pauliani
Stygiocaris stylifera
Stygiocaris lancifera
Halocaridina rubra
Halocaridinides fowleri
Acetes chinensis
Marsupenaeus japonicus
Litopenaeus stylirostris
Euphausia pacifica
0,99
0,91
0,99
0,79
0,98
0,97
0.6

O

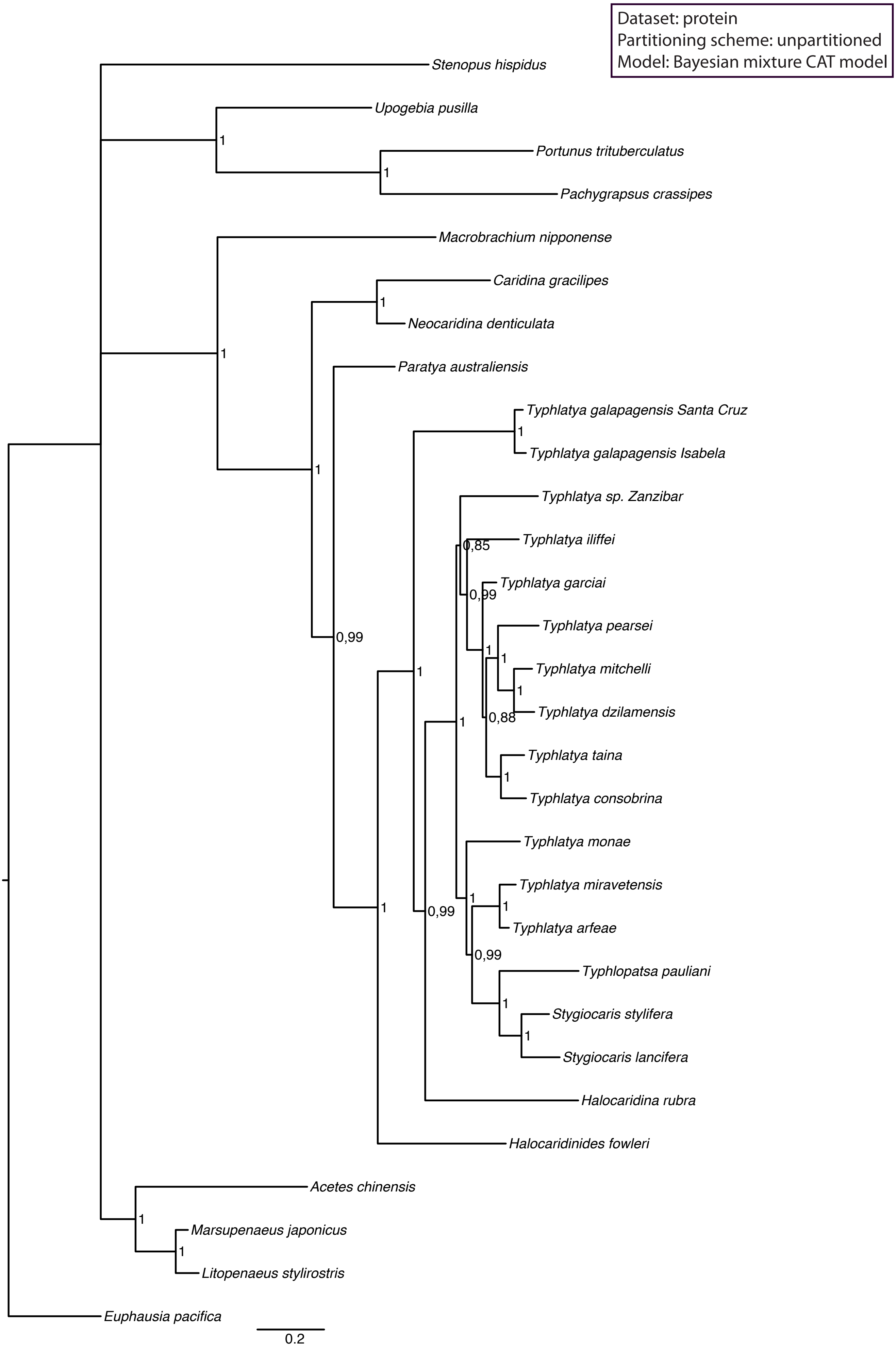
Dataset: protein
Partitioning scheme: unpartitioned
Model: Bayesian mixture CAT model
Stenopus hispidus
Upogebia pusilla
Portunus trituberculatus
Pachygrapsus crassipes
Macrobrachium nipponense
Caridina gracilipes
Neocaridina denticulata
Paratya australiensis
Typhlatya galapagensis Santa Cruz
Typhlatya galapagensis Isabela
Typhlatya sp. Zanzibar
Typhlatya iliffei
Typhlatya garciai
Typhlatya pearsei
Typhlatya mitchelli
Typhlatya dzilamensis
Typhlatya taina
Typhlatya consobrina
Typhlatya monae
Typhlatya miravetensis
Typhlatya arfeae
Typhlopatsa pauliani
Stygiocaris stylifera
Stygiocaris lancifera
Halocaridina rubra
Halocaridinides fowleri
Acetes chinensis
Marsupenaeus japonicus
Litopenaeus stylirostris
Euphausia pacifica
0,85
0,99
0,88
0,99
0,99
0,99
0.2

p

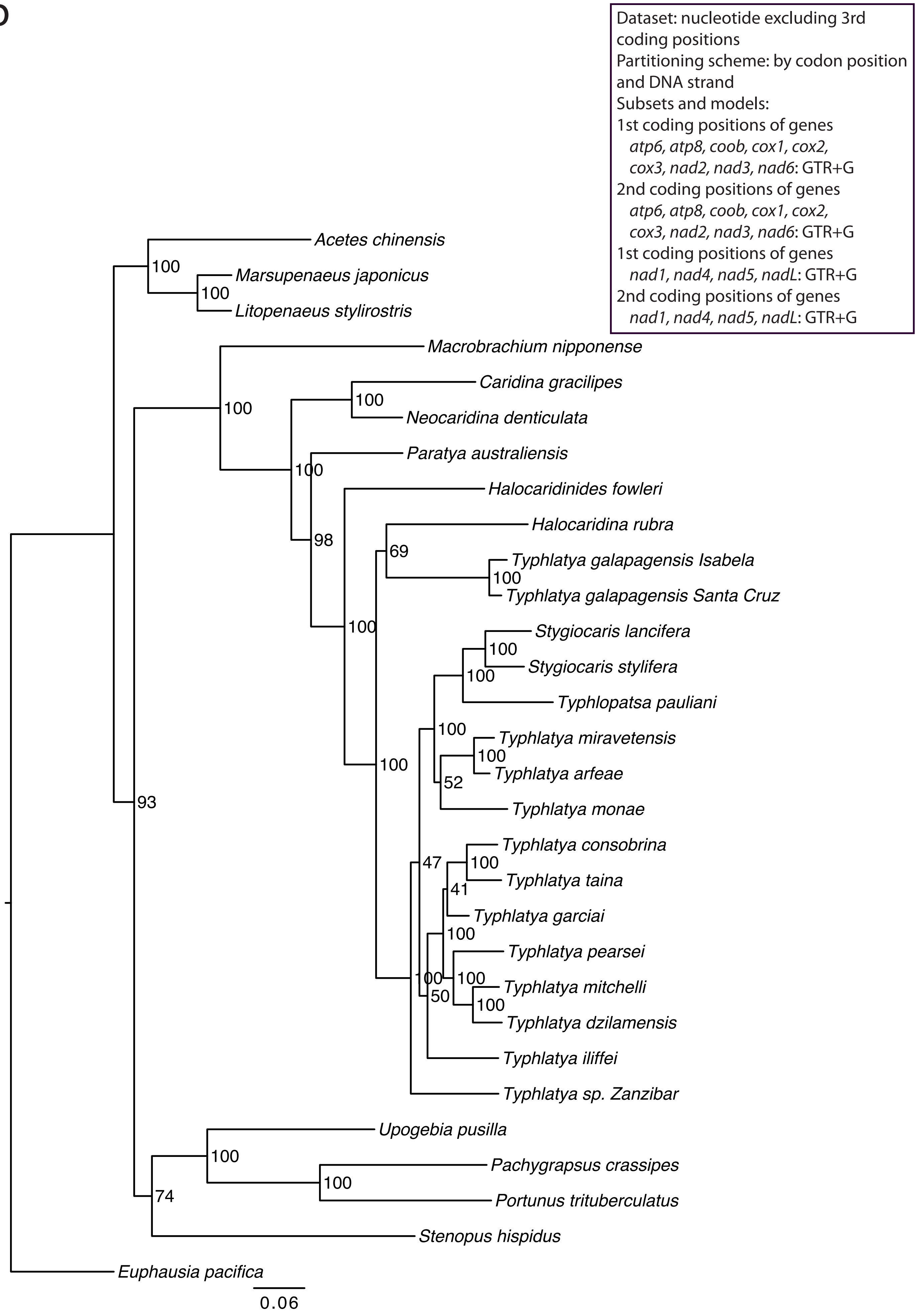
Dataset: nucleotide excluding 3rd coding positions
Partitioning scheme: by codon position and DNA strand
Subsets and models:
1st coding positions of genes *atp6, atp8, coob, cox1, cox2, cox3, nad2, nad3, nad6*: GTR+G
2nd coding positions of genes *atp6, atp8, coob, cox1, cox2, cox3, nad2, nad3, nad6*: GTR+G
1st coding positions of genes *nad1, nad4, nad5, nadL*: GTR+G
2nd coding positions of genes *nad1, nad4, nad5, nadL*: GTR+G
Acetes chinensis
Marsupenaeus japonicus
Litopenaeus stylirostris
Macrobrachium nipponense
Caridina gracilipes
Neocaridina denticulata
Paratya australiensis
Halocaridinides fowleri
Halocaridina rubra
Typhlatya galapagensis Isabela
Typhlatya galapagensis Santa Cruz
Stygiocaris lancifera
Stygiocaris stylifera
Typhlopatsa pauliani
Typhlatya miravetensis
Typhlatya arfeae
Typhlatya monae
Typhlatya consobrina
Typhlatya taina
Typhlatya garciai
Typhlatya pearsei
Typhlatya mitchelli
Typhlatya dzilamensis
Typhlatya iliffei
Typhlatya sp. Zanzibar
Upogebia pusilla
Pachygrapsus crassipes
Portunus trituberculatus
Stenopus hispidus
Euphausia pacifica
0.06

q

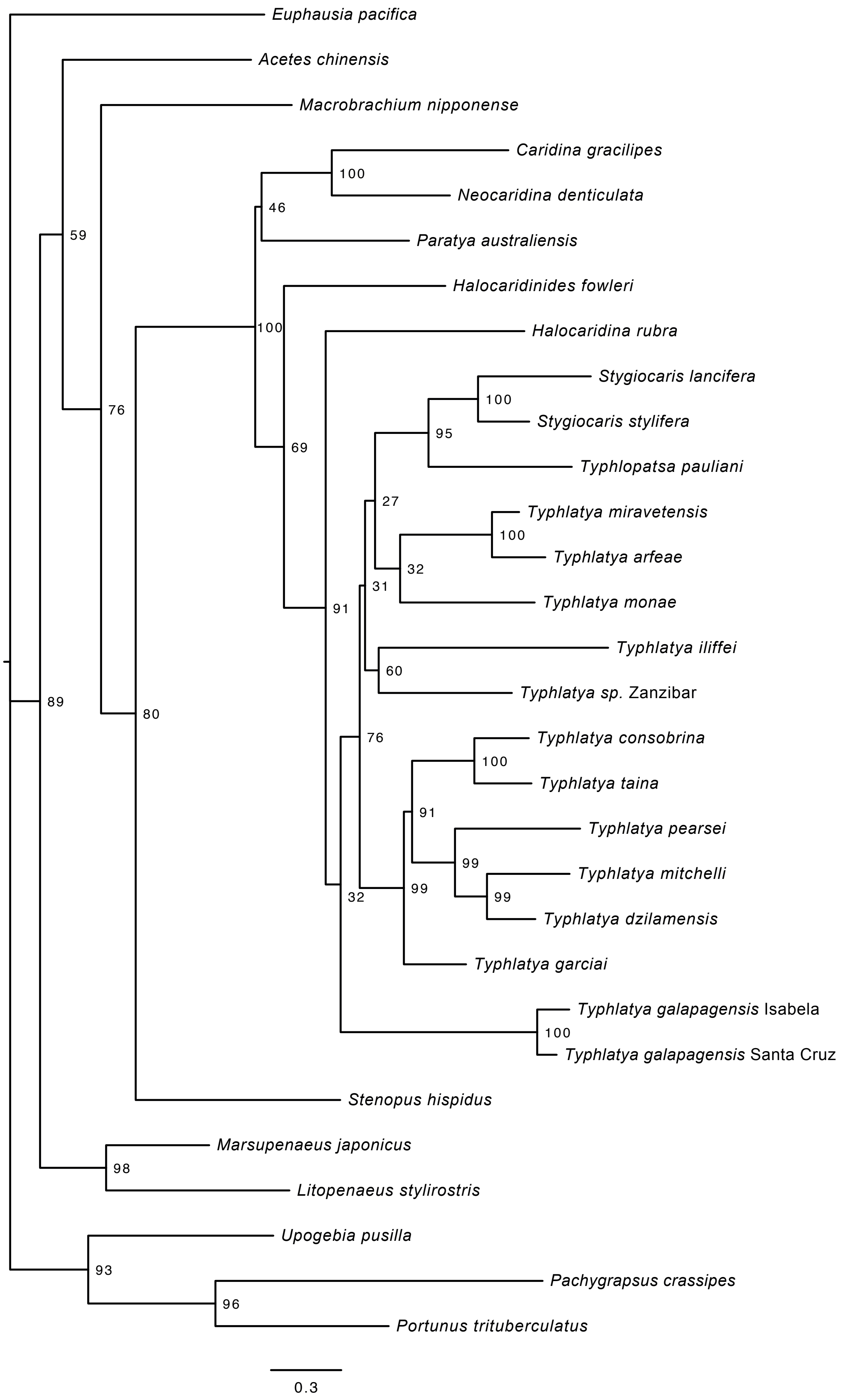

Euphausia pacifica
Acetes chinensis
Macrobrachium nipponense
Caridina gracilipes
100
Neocaridina denticulata
46
Paratya australiensis
59
Halocaridinides fowleri
100
Halocaridina rubra
Stygiocaris lancifera
100
Stygiocaris stylifera
76
95
69
Typhlopatsa pauliani
27
Typhlatya miravetensis
100
Typhlatya arfeae
32
31
Typhlatya monae
91
Typhlatya iliffei
60
Typhlatya sp. Zanzibar
89
80
Typhlatya consobrina
76
100
Typhlatya taina
91
Typhlatya pearsei
99
Typhlatya mitchelli
99
32
99
Typhlatya dzilamensis
Typhlatya garciai
Typhlatya galapagensis Isabela
100
Typhlatya galapagensis Santa Cruz
Stenopus hispidus
Marsupenaeus japonicus
98
Litopenaeus stylirostris
Upogebia pusilla
93
Pachygrapsus crassipes
96
Portunus trituberculatus
0.3

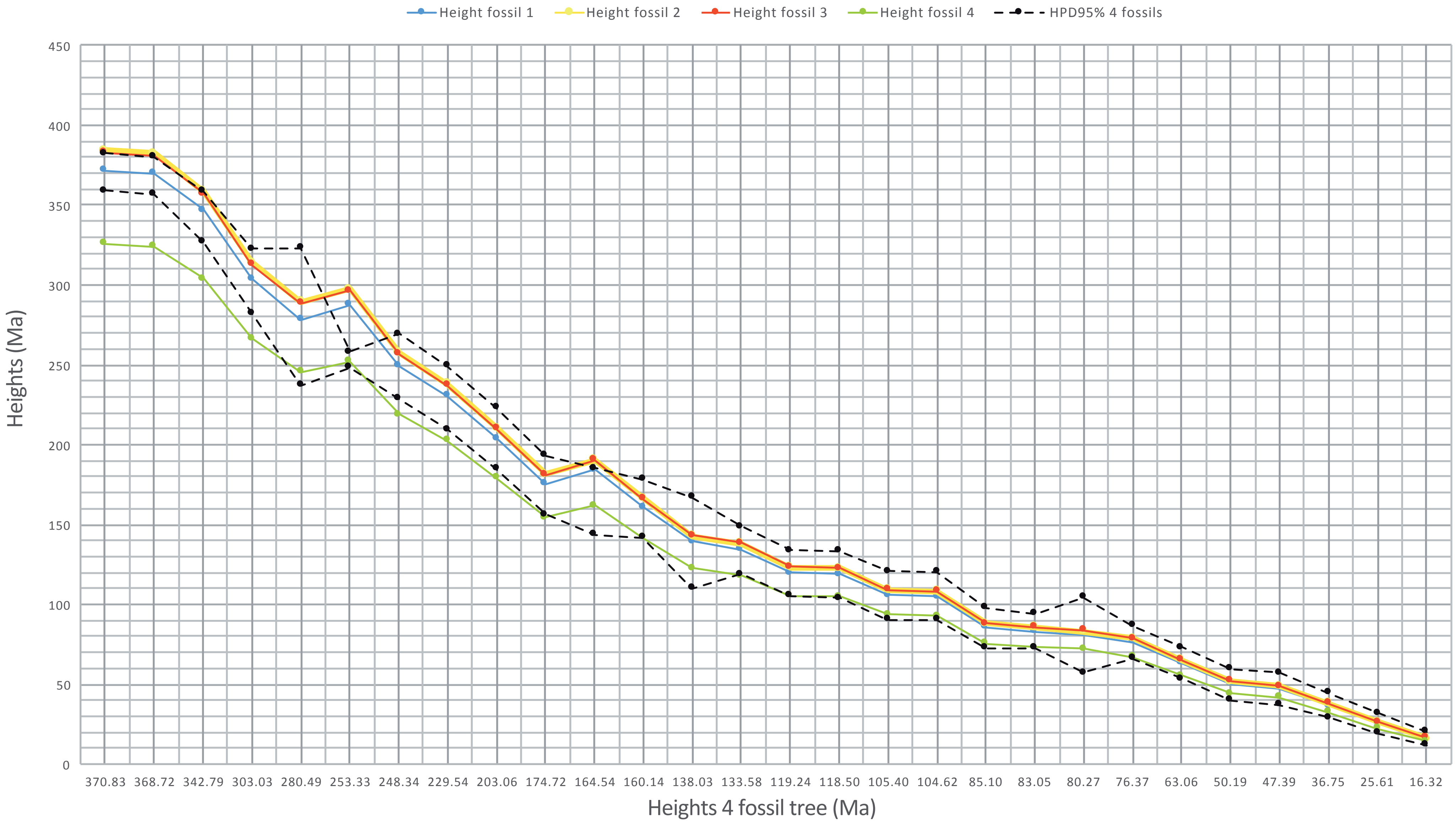

Supplementary Figure 2. Results of the fossil cross-validation test. Dating analyses were independently performed using single-fossil calibrations (colored dots and lines) and using the four fossils at a time (HPD95% shown in dashed lines). The inferred ages from the individual fossil analyses were largely congruent with the four fossil calibration strategy followed in this study.

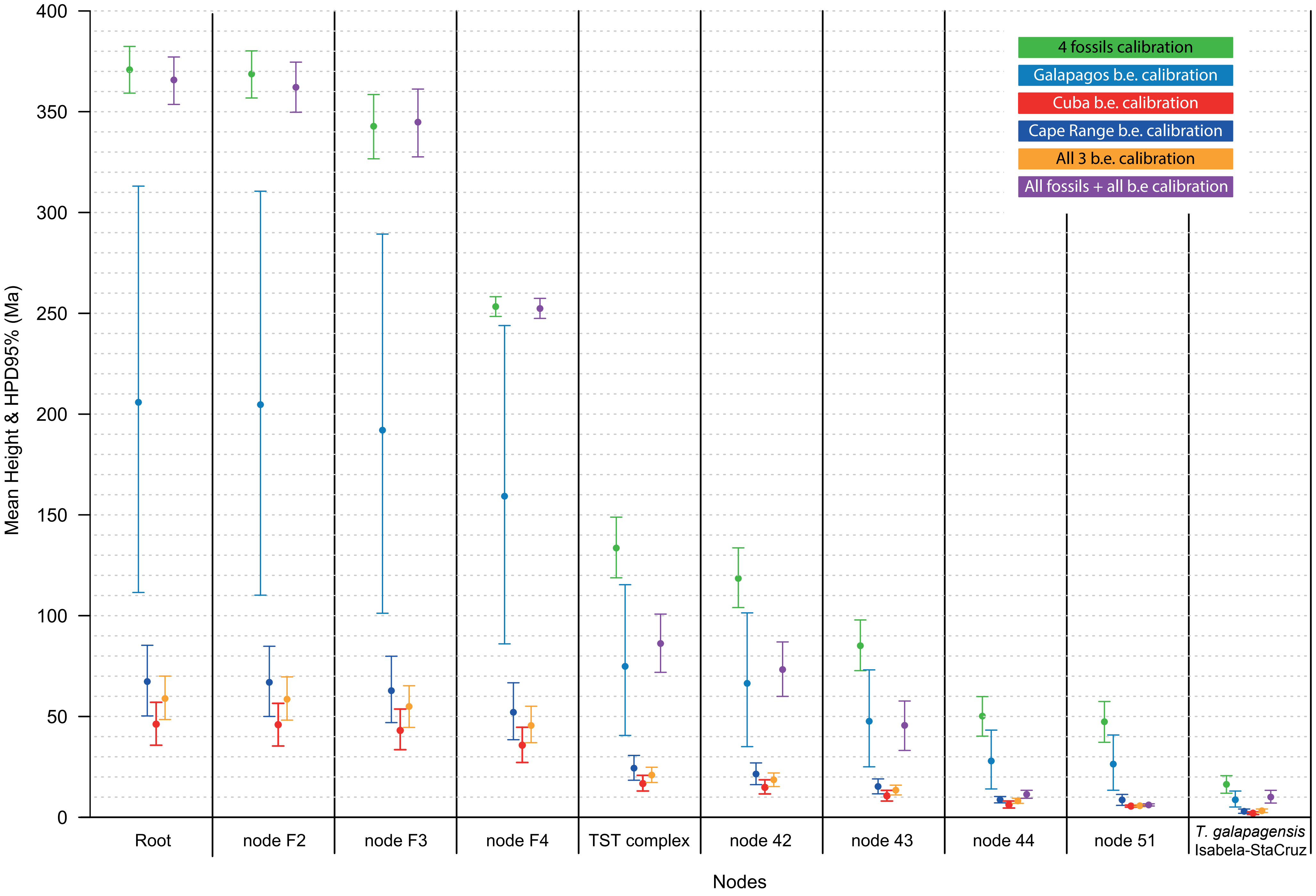

Supplementary Figure 3. Node age comparison from different calibration schemes. Dots represent mean height values and error bars correspond to their respective 95% highest posterior density (HPD) intervals. Nodes are coded according to Figure 2 in the main text.

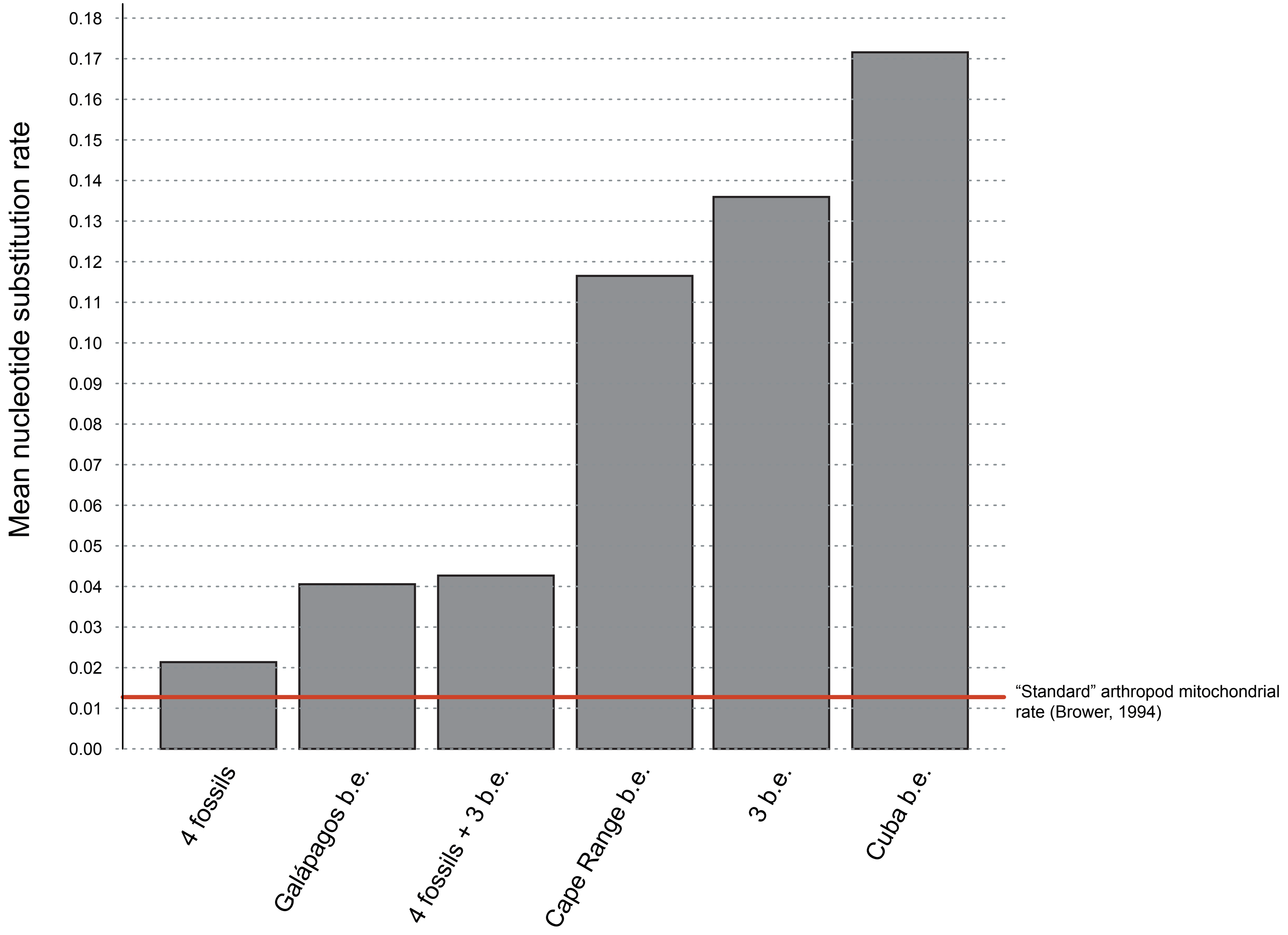

Supplementary Figure 4. Mean nucleotide substitution rates across the tree derived from reanalysing the dataset under different calibration strategies (see Methods in main text). The horizontal red bar correspond to the “standard” arthropod mitochondrial rate from Brower (1994). b.e. = biogeographical event.

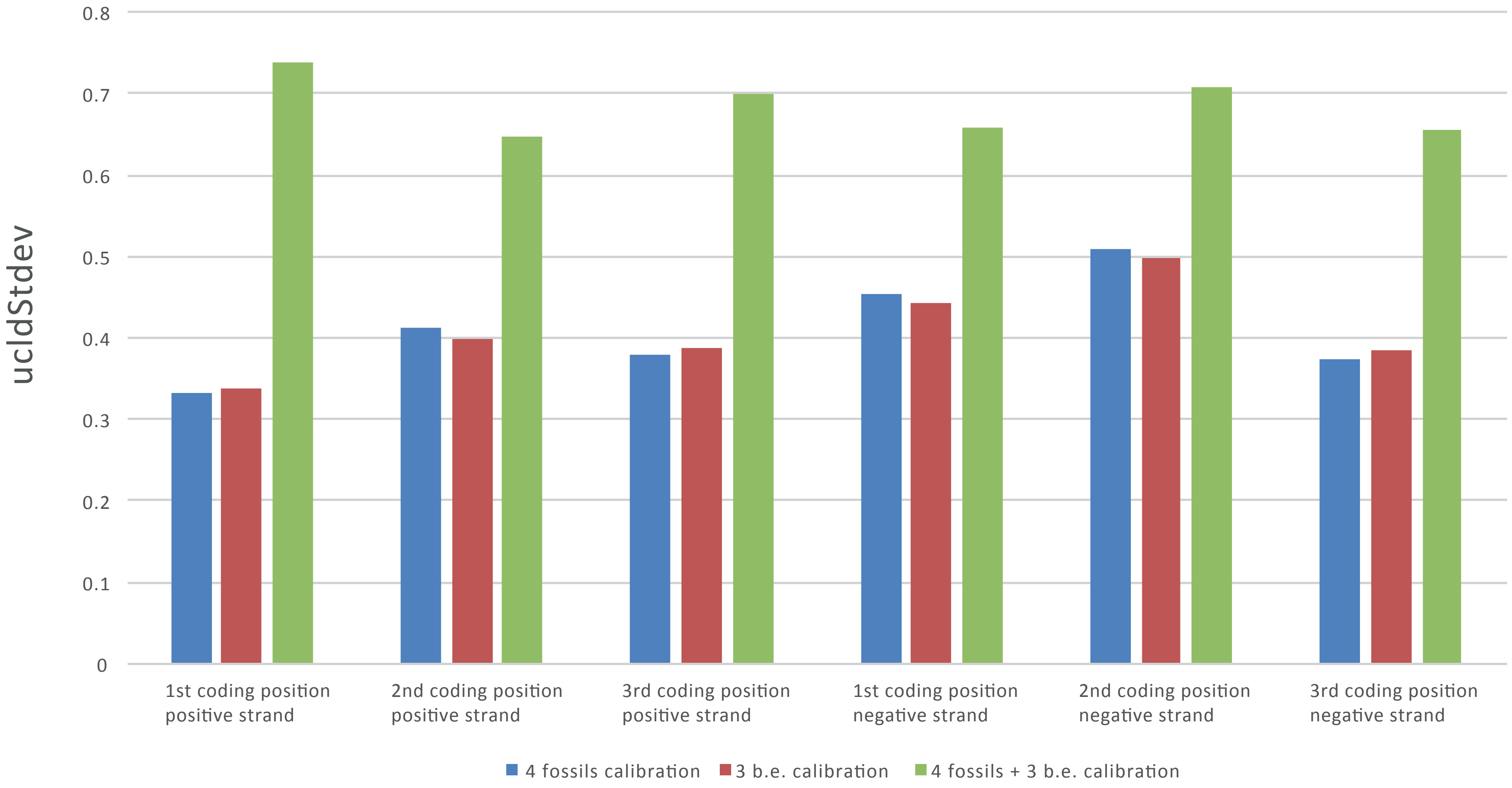

Supplementary Figure 5. Values of the ucldStdev parameter derived from analysing the dataset under three different calibration schemes (see Methods in main text). b.e.= biogeographical event.

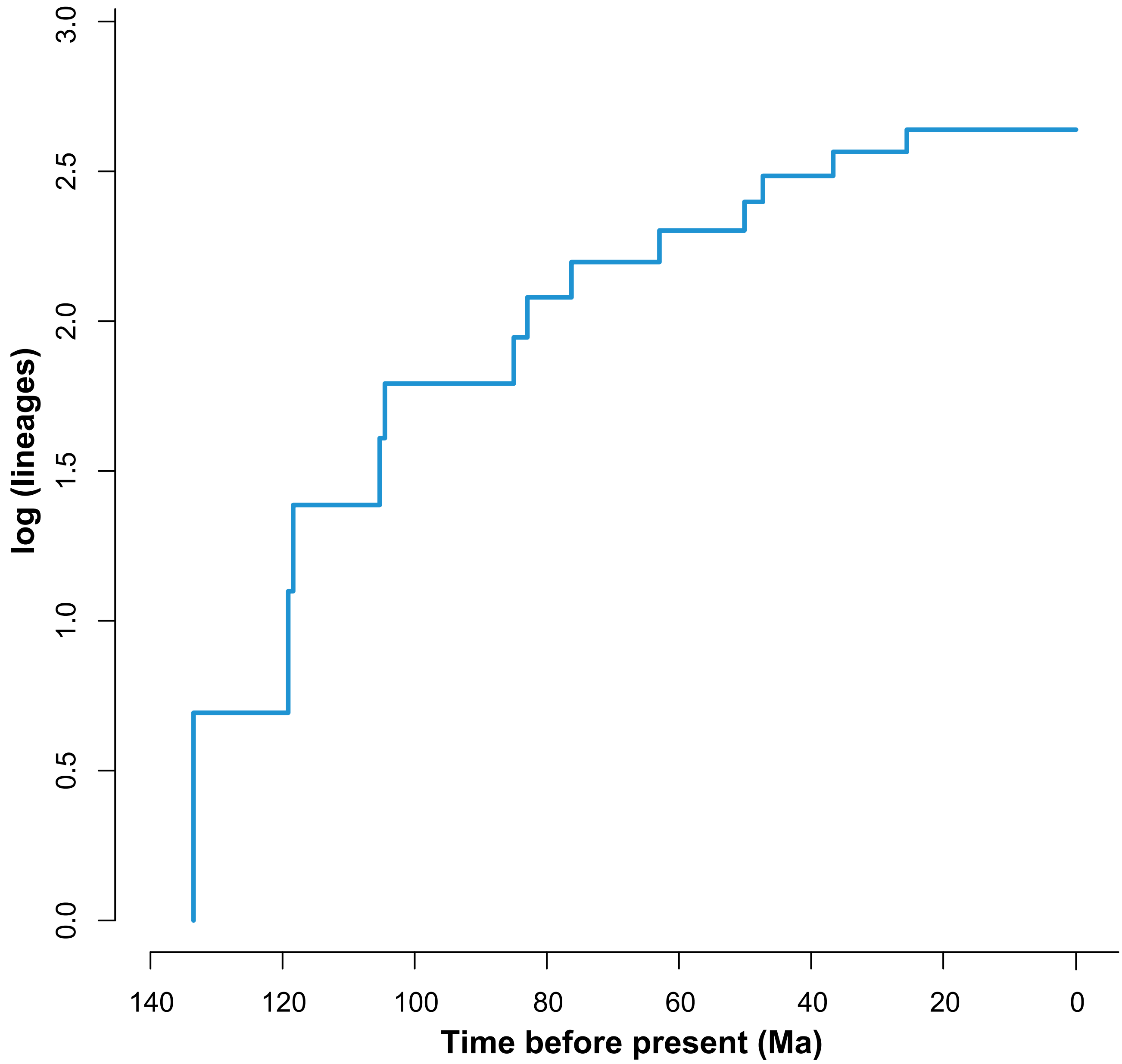

Supplementary Figure 6. Lineages through time plot of the TST complex phylogeny.

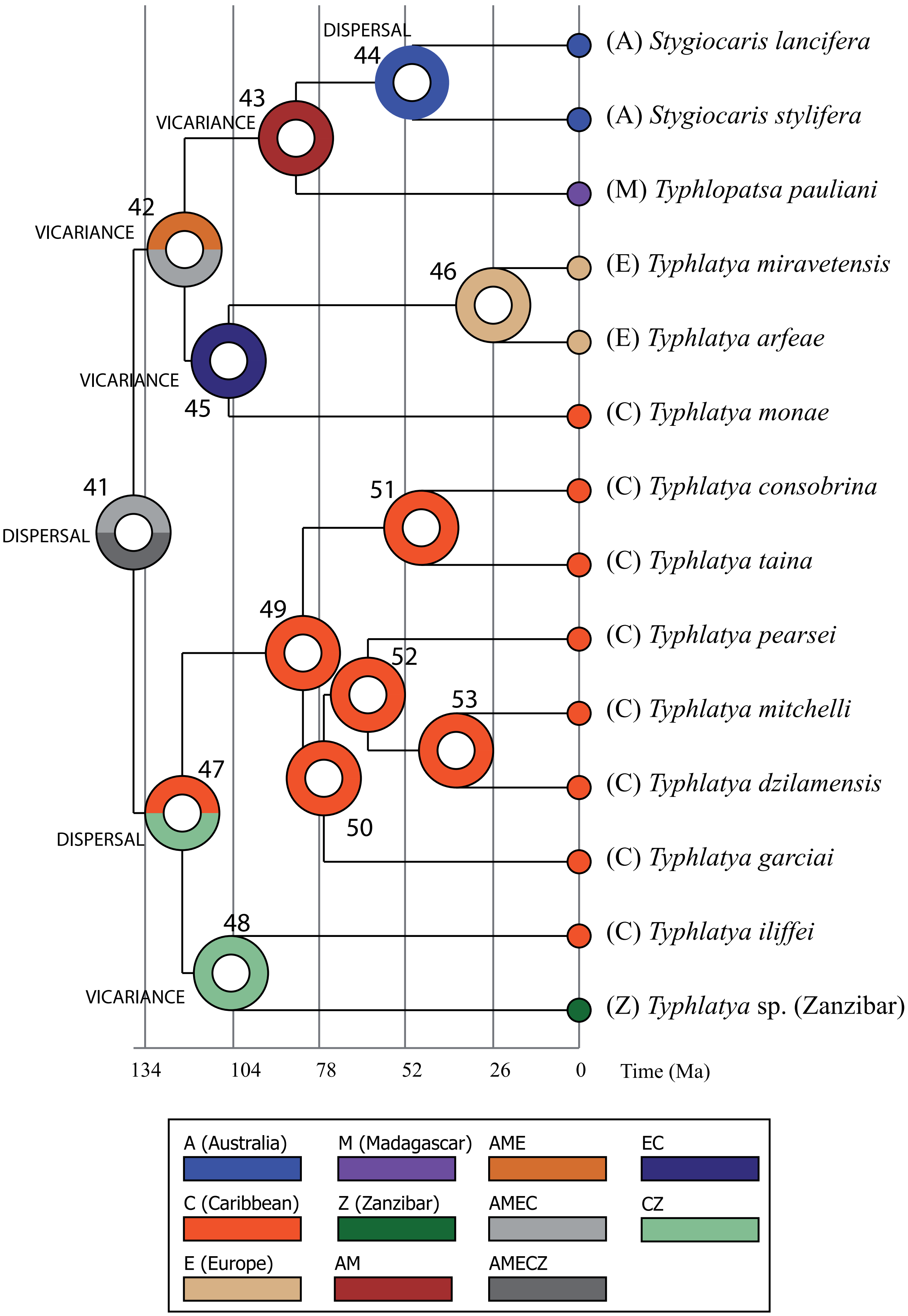

Supplementary Figure 7. Reconstruction of the biogeographic history of the TST complex inferred in S-Diva. Nodes are numbered according to Fig. 1 for their discussion in the main text. Colored rings refer to the ancestral distribution inferred for each node. Dispersal and vicariance events derived from the analysis are also indicated.

		All positions					First coding positions					Second coding positions					Third coding positions				
**Gene**	**Length (bp)**	**%T**	**%C**	**%A**	**%G**	**%A+T**	**%T**	**%C**	**%A**	**%G**	**%A+T**	**%T**	**%C**	**%A**	**%G**	**%A+T**	**%T**	**%C**	**%A**	**%G**	**%A+T**
atp6	672	35.1	22.6	28.8	13.6	63.9	25.3	23.6	31.4	19.6	56.7	46.1	25.3	14.4	14.2	60.5	33.9	18.7	40.5	6.9	74.4
atp8	150	38.8	20.4	33.6	7.2	72.4	36.3	21.1	35.3	7.3	71.6	45.7	21.1	24.3	9	70	34.5	19	41.3	5.3	75.8
coob	1131	34.4	23.7	27.9	14	62.3	26.8	22.9	26.6	23.7	53.4	43.1	24.3	19.4	13.3	62.5	33.4	23.9	37.6	5	71
cox1	1524	33.1	22.3	27.7	17	60.8	23.7	20.3	27.3	28.7	51	41.1	25.2	17.4	16.3	58.5	34.4	21.4	38.4	5.8	72.8
cox2	681	31.7	22.2	30.9	15.2	62.6	24	21.9	28.8	25.2	52.8	38.9	22.8	23.3	15.1	62.2	32.2	21.8	40.6	5.4	72.8
cox3	780	33.3	23	27.8	15.8	61.1	28.4	22.2	24.4	25	52.8	37.5	24.7	21	16.8	58.5	34	22.2	38	5.8	72
nad1	930	44	11.8	22.5	21.7	66.5	37.3	11.7	24	26.9	61.3	46.7	17.9	17.8	17.6	64.5	48	5.6	25.8	20.6	73.8
nad2	918	37.6	24.3	27.3	10.8	64.9	32.4	23.2	28.7	15.6	61.1	46.7	28.5	15.3	9.5	62	33.6	21.3	37.8	7.3	71.4
nad3	348	38.2	21.1	27.4	13.4	65.6	28.8	18.9	30.3	22	59.1	48.1	23.5	16.6	11.8	64.7	37.6	20.9	35.2	6.2	72.8
nad4	1329	42.7	10.9	23.7	22.7	66.4	35.4	11.8	27	25.8	62.4	47.1	15.1	15.3	22.5	62.4	45.5	5.7	28.9	20	74.4
nad5	1719	41.2	11.6	25.2	22	66.4	33.3	9.8	28.5	28.4	61.8	44.4	18.7	18.1	18.9	62.5	45.9	6.4	29	18.7	74.9
nad6	474	39.1	22.7	29.3	8.9	68.4	30	19	35.6	15.4	65.6	52.1	28.4	13.2	6.2	65.3	35.1	20.7	39	5.1	74.1
nadL	297	45.3	9.7	21.9	23.1	67.2	37.9	10.5	25	26.7	62.9	50.7	13.3	13.8	22.2	64.5	47.3	5.2	27	20.5	74.3
Total	10953	38.0	18.9	27.2	15.8	65.3	30.7	18.2	28.7	22.3	59.4	45.2	22.2	17.7	14.9	62.9	38.1	16.4	35.3	10.2	73.4

Supplementary Table 1. Sequence statistics of the mitochondrial protein coding genes used in this study.

Partitioning scheme	Log Likelihood	Free parameters	Number of Partitions	BIC
by codon position and DNA strand	-180890.5	393	6	365436.42
by codon position, DNA strand and nucleotide substitution rate	-180063.7	583	9	365550.06
by codon position	-185023.8	198	3	371889.35
(1st + 2nd) and 3rd codon positions	-186187.8	132	2	373603.42
by gene and codon position	-178390.2	2505	39	380080.37
by gene	-186939.6	854	13	381822.52
unpartitioned	-218147.5	61	1	436862.31

Supplementary Table 2. Results of partitioning analysis.

Footnote: The best partitioning scheme (i.e., the one with the lowest BIC score) for the nucleotide-based analyses consisted on subdividing the dataset by codon position and coding DNA strand. (BIC: Bayesian information criterion).

Dataset	Partitioning scheme	Log likelihood	Free parameters	Number of Partitions	BIC
Nucleotide under codon-based substitution models	By DNA strand	-175034,586	248	2	352103,455
	unpartitioned	-179699,702	124	1	360416,545
Protein translation of the original nucleotide dataset	PartitionFinder best scheme	-63477,713	234	3	128874,871
	by gene	-68013,315	861	13	143089,204
	by DNA strand	-69022,218	156	2	139324,066
	unpartitioned	-69577,244	78	1	139794,303

Supplementary Table 3. Results of partitioning analysis in the nucleotide dataset through the implementation of codon-based substitution models and also at the protein level.

Footnote: Detailed information on the partition schemes and their respective evolutionary models are shown in Supplementary Figure 1.

Partition scheme	Number of partitions	Substitution models	Number of clocks	Clock model	Diversification model	Marginal Likelihood	Bayes Factors	AICM	Δ AICM
Cod. pos. & strand	6	1st & 2nd cod. pos.=GTR+G<br>3rd cod. pos.=TrN+G	6	UCLN	Yule	-175140.241	0	349510.441	0
Cod. pos. & strand	6	1st & 2nd cod. pos.=GTR+G<br>3rd cod. pos.=TrN+G	6	STC	Yule	-175633.759	987.035	350743.378	1232.937
Cod. pos. & strand	6	1st & 2nd cod. pos.=GTR+G<br>3rd cod. pos.=TrN+G	6	RLC	Yule	n.a.	n.a.	350064.703	554.262
Cod. pos. & strand	6	1st & 2nd cod. pos.=GTR+G<br>3rd cod. pos.=TrN+G	3 (Cod. pos.)	UCLN	Yule	-175149.464	18.446	349594.334	83.893
Cod. pos. & strand	6	1st & 2nd cod. pos.=GTR+G<br>3rd cod. pos.=TrN+G	3 (Cod. pos.)	STC	Yule	-175673.017	1065.552	350829.560	1319.119
Cod. pos. & strand	6	1st & 2nd cod. pos.=GTR+G<br>3rd cod. pos.=TrN+G	3 (Cod. pos.)	RLC	Yule	n.a.	n.a.	349877.430	366.989
Cod. pos. & strand	6	1st & 2nd cod. pos.=GTR+G<br>3rd cod. pos.=TrN+G	2 (1st+2nd / 3rd)	UCLN	Yule	-175379.234	477.985	350110.637	600.196
Cod. pos. & strand	6	1st & 2nd cod. pos.=GTR+G<br>3rd cod. pos.=TrN+G	2 (1st+2nd / 3rd)	STC	Yule	-175922.646	1564.809	351335.078	1824.637
Cod. pos. & strand	6	1st & 2nd cod. pos.=GTR+G<br>3rd cod. pos.=TrN+G	1	UCLN	Yule	-178197.852	6115.220	355778.754	6268.313
Cod. pos. & strand	6	1st & 2nd cod. pos.=GTR+G<br>3rd cod. pos.=TrN+G	1	STC	Yule	-178558.975	6837.467	356593.009	7082.568
Cod. pos.	3	1st & 2nd cod. pos.=GTR+G<br>3rd cod. pos.=TrN+G	3 (Cod. pos.)	UCLN	Yule	-179059.062	7837.640	357506.423	7995.982
Cod. pos.	3	1st & 2nd cod. pos.=GTR+G<br>3rd cod. pos.=TrN+G	3 (Cod. pos.)	RLC	Yule	n.a.	n.a.	357797.378	8286.937
Cod. pos. & strand	6	1st & 2nd cod. pos.=GTR+G<br>3rd cod. pos.=TrN+G	6	best Yule analysis (6 clocks UCLN)	Birth-Death	-175138.345	-3.792	349506.576	-3.865

Supplementary Table 4. Summary of the model comparison analyses performed in BEAST.

Footnote: The scheme with the lower Δ Bayes factor consisted on subdividing the data set into 6 partitions with 6 independent UCLN clocks. Under this scheme both Yule and Birth-Death diversification models resulted in Bayes factor values differing in less than 10 units; therefore we chose the Yule model because it was the one that includes fewer parameters. (UCLN: uncorrelated log-normal clock model. STC: strict clock model. RLC: random local clock model. Cod. pos.: Coding position. AICM: Akaike information criterion through MCMC).

		node30			node31			node32			node33		
	Partitioning scheme/clock number and clock model/diversification model	node height	HPD95 height	pp	node height	HPD95 height	pp	node height	HPD95 height	pp	node height	HPD95 height	pp
Analyzing all positions	6 partitions/6 UCLN/Yule	370.83	359.23-382.40	1	280.49	237.23-322.98	1	80.27	57.30-104.71	1	368.72	356.81-380.20	1
	6 partitions/6 UCLN/Birth-Death	370.84	359.26-382.84	1	280.39	235.71-321.01	1	80.27	58.36-104.39	1	368.71	357.22-381.16	1
	6 partitions/3 UCLN (by cod. pos.)/Yule	373.48	361.62-385.84	1	285.16	232.79-333.39	1	82.1	51.61-114.74	1	370.38	358.18-383.13	1
	6 partitions/3 RLC (by cod. pos.)/Yule	374.65	361.66-387.92	1	312.25	281.62-348.31	1	173.69	100.49-219.98	1	362.51	348.71-376.44	1
	6 partitions/6 RLC /Yule	373.31	359.82-387.52	1	314.63	272.18-361.43	1	120.75	88.58-158.42	1	355.57	340.17-371.21	1
Excluding 3rd coding positions	4 partitions/4 UCLN/Yule	372.71	361.03-384.87	1	236.19	184.00-287.31	1	46.13	31.58-61.76	1	370.46	358.39-382.52	1
	4 partitions/4 UCLN/Birth-Death	372.81	360.93-384.66	1	237.08	184.07-289.90	1	46.18	31.60-61.60	1	370.56	358.73-382.81	1
	4 partitions/2 UCLN (by cod. pos.)/Yule	374.81	362.71-386.91	1	227.56	166.97-288.88	1	47.91	28.23-69.96	1	371.63	359.31-384.25	1
	4 partitions/4 RLC/Yule	373.11	360.56-386.21	1	288.15	252.83-327.06	1	97.59	80.08-116.10	1	361.06	347.64-374.32	1
	4 partitions/2 RLC (by cod. pos.)/Yule	376.41	364.09-388.98	1	286.64	242.99-322.75	1	101.04	80.71-122.56	1	369.25	355.67-382.47	1

		node34			node35			node36			node37		
		node height	HPD95 height	pp	node height	HPD95 height	pp	node height	HPD95 height	pp	node height	HPD95 height	pp
Analyzing all positions	6 partitions/6 UCLN/Yule	303.03	282.36-322.54	1	248.34	228.62-269.19	1	229.54	209.72-249.13	1	138.03	110.07-167.26	1
	6 partitions/6 UCLN/Birth-Death	303.22	283.10-323.17	1	248.37	228.00-267.46	1	229.59	210.48-248.93	1	138.49	109.44-168.13	1
	6 partitions/3 UCLN (by cod. pos.)/Yule	304.49	279.76-329.12	1	252.07	227.30-277.58	1	230.85	206.11-255.29	1	141.76	101.92-180.50	1
	6 partitions/3 RLC (by cod. pos.)/Yule	274.35	258.21-290.34	1	212.34	195.40-229.33	1	193.47	176.94-209.25	1	136.44	122.36-151.59	1
	6 partitions/6 RLC /Yule	272.91	255.83-290.92	1	215.74	198.56-233.57	1	196.31	179.03-213.2	1	139.35	124.74-154.88	1
Excluding 3rd coding positions	4 partitions/4 UCLN/Yule	297.04	273.18-320.38	1	231.05	208.37-253.66	1	211.66	190.09-233.48	1	118.22	87.14-150.91	1
	4 partitions/4 UCLN/Birth-Death	296.74	272.96-319.90	1	230.57	208.22-253.36	1	211.18	189.56-233.03	1	117.64	86.54-150.24	1
	4 partitions/2 UCLN (by cod. pos.)/Yule	297.56	267.76-327.09	1	232.98	203.22-262.68	1	211.14	183.05-239.96	1	121.51	80.10-165.61	1
	4 partitions/4 RLC/Yule	271.26	255.88-286.61	1	195.8	183.49-207.88	1	n.a.	n.a.	n.a.	84.23	74.72-94.08	1
	4 partitions/2 RLC (by cod. pos.)/Yule	261.72	244.35-279.19	1	154.41	140.79-168.46	1	125.36	111.3-139.69	1	71.46	59.23-83.90	1

		node38			node40			node41			node42		
		node height	HPD95 height	pp	node height	HPD95 height	pp	node height	HPD95 height	pp	node height	HPD95 height	pp
Analyzing all positions	6 partitions/6 UCLN/Yule	203.06	184.82-222.98	1	174.72	156.52-193.10	1	133.58	118.79-148.89	1	118.5	104.04-133.66	1
	6 partitions/6 UCLN/Birth-Death	203.18	184.59-221.50	1	174.86	157.37-192.56	1	133.58	119.00-147.84	1	118.51	104.60-132.86	1
	6 partitions/3 UCLN (by cod. pos.)/Yule	204.09	179.98-227.80	1	175.76	153.93-199.20	1	133.94	115.55-152.68	1	118.67	101.44-138.24	1
	6 partitions/3 RLC (by cod. pos.)/Yule	165.29	150.88-179.69	1	140.96	127.76-153.89	1	103.3	92.91-113.43	1	93.67	83.83-103.24	1
	6 partitions/6 RLC /Yule	169.45	154.28-184.40	1	144.08	130.95-157.34	1	105.19	94.80-115.67	1	92.74	81.43-104.14	1
Excluding 3rd coding positions	4 partitions/4 UCLN/Yule	181.78	161.94-201.94	1	154.58	136.43-172.49	1	117.66	102.61-132.99	1	94.42	81.93-107.16	1
	4 partitions/4 UCLN/Birth-Death	181.25	161.55-201.61	1	154	136.16-172.08	1	116.98	101.92-132.79	1	94.39	81.68-107.20	1
	4 partitions/2 UCLN (by cod. pos.)/Yule	179.77	154.01-206.15	1	151.88	129.12-175.36	1	113.88	95.89-132.36	1	96.31	79.82-113.40	1
	4 partitions/4 RLC/Yule	173.04	161.89-184.15	1	141.88	131.79-152.05	0.91	100.54	92.44-108.64	1	75.83	68.44-83.35	1
	4 partitions/2 RLC (by cod. pos.)/Yule	101.96	89.17-115.20	1	85.07	73.93-96.40	1	60.11	52.00-68.52	1	43.64	37.30-50.20	1

Supplementary Table 5. Estimated age, higher posterior densities 95% and posterior probability values for each node in the best trees according to Bayes factor scores obtained under different clock and speciation models in the analyses performed in BEAST2. Results derived from both the original nucleotide dataset (6 partitions) and the dataset without 3rd coding positions (4 partitions) are shown. Node number refer to Fig. 1. (UCLN: uncorrelated log-normal clock model, RLC: Random local clocks model, cod. pos.: coding positions). Continues in the next page.

		node43			node44			node45			node46		
		node height	HPD95 height	pp	node height	HPD95 height	pp	node height	HPD95 height	pp	node height	HPD95 height	pp
Analyzing all positions	6 partitions/6 UCLN/Yule	85.1	72.74-97.88	1	50.19	40.26-59.87	1	105.4	90.63-120.91	0.99	25.61	19.38-32.28	1
	6 partitions/6 UCLN/Birth-Death	85.18	73.06-97.37	1	50.23	40.58-60.15	1	105.41	90.61-120.08	0.99	25.52	19.10-32.20	1
	6 partitions/3 UCLN (by cod. pos.)/Yule	84.04	68.41-100.38	1	49.66	36.98-62.84	1	105.26	87.53-125.03	0.98	25.86	17.56-34.87	1
	6 partitions/3 RLC (by cod. pos.)/Yule	66.15	58.50-73.77	1	39.08	34.11-44.15	1	84.18	74.80-93.53	0.99	28.97	22.18-35.12	1
	6 partitions/6 RLC /Yule	65.08	56.34-74.72	1	36.77	31.80-42.08	1	81.49	70.59-92.56	0.99	22.55	17.09-27.25	1
Excluding 3rd coding positions	4 partitions/4 UCLN/Yule	70	58.44-81.73	1	45.45	35.41-55.78	1	82.28	68.87-95.67	0.99	25.19	17.57-33.52	1
	4 partitions/4 UCLN/Birth-Death	69.97	58.40-81.76	1	45.46	35.38-55.76	1	82.25	68.82-96.01	0.98	25.14	17.57-33.30	1
	4 partitions/2 UCLN (by cod. pos.)/Yule	70.4	55.13-85.85	1	45.07	31.71-58.94	1	83.84	66.52-101.85	0.92	25.34	15.03-36.44	1
	4 partitions/4 RLC/Yule	57.65	51.22-64.31	1	35.89	30.85-41.16	1	64.68	57.84-71.40	0.99	20.02	17.05-23.06	1
	4 partitions/2 RLC (by cod. pos.)/Yule	29.1	23.38-35.16	1	18.15	14.31-22.25	1	43.64	37.30-50.21	1	12.63	10.32-15.05	1

		node47			node48			node49			node50		
		node height	HPD95 height	pp	node height	HPD95 height	pp	node height	HPD95 height	pp	node height	HPD95 height	pp
Analyzing all positions	6 partitions/6 UCLN/Yule	119.24	105.21-133.88	0.97	104.62	90.63-120.61	0.96	83.05	72.78-94.00	1	76.37	66.09-86.65	0.98
	6 partitions/6 UCLN/Birth-Death	119.16	105.05-132.67	0.97	104.55	90.39-119.28	0.96	82.95	72.56-93.59	1	76.37	66.27-86.62	0.97
	6 partitions/3 UCLN (by cod. pos.)/Yule	119.69	102.58-137.70	1	104.41	86.49-123.22	1	83.75	70.23-97.59	1	76.34	63.39-89.59	1
	6 partitions/3 RLC (by cod. pos.)/Yule	95.31	85.51-105.35	0.99	n.a.	n.a.	n.a.	66.98	59.31-74.53	1	61.4	53.36-69.1	0.95
	6 partitions/6 RLC /Yule	97.95	88.38-108.63	0.87	n.a.	n.a.	n.a.	68.44	60.88-76.50	1	64.65	57.67-71.57	0.6
Excluding 3rd coding positions	4 partitions/4 UCLN/Yule	117.66	102.61-132.99	1	n.a.	n.a.	n.a.	72.55	61.56-83.82	1	n.a.	n.a.	n.a.
	4 partitions/4 UCLN/Birth-Death	116.98	101.92-132.79	1	n.a.	n.a.	n.a.	72.39	61.01-83.68	1	n.a.	n.a.	n.a.
	4 partitions/2 UCLN (by cod. pos.)/Yule	103.41	86.42-121.09	0.9	88.86	70.54-107.32	0.9	74.01	60.08-88.61	1	69.29	55.4-83.62	0.37
	4 partitions/4 RLC/Yule	100.54	92.44-108.64	1	n.a.	n.a.	n.a.	61.57	55.71-67.52	1	n.a.	n.a.	n.a.
	4 partitions/2 RLC (by cod. pos.)/Yule	60.11	52.00-68.52	1	n.a.	n.a.	n.a.	38.72	33.20-44.23	1	n.a.	n.a.	n.a.

		node51			node52			node53			node54		
		node height	HPD95 height	pp	node height	HPD95 height	pp	node height	HPD95 height	pp	node height	HPD95 height	pp
Analyzing all positions	6 partitions/6 UCLN/Yule	47.39	37.18-57.44	1	63.06	53.59-72.98	1	36.75	29.39-44.63	1	16.32	11.95-20.63	1
	6 partitions/6 UCLN/Birth-Death	47.42	37.40-57.84	1	62.98	53.28-72.38	1	36.72	29.38-44.65	1	16.26	12.14-20.60	1
	6 partitions/3 UCLN (by cod. pos.)/Yule	47.07	33.95-60.07	1	62.12	49.76-74.31	1	35.96	26.22-45.69	1	16.42	10.84-22.34	1
	6 partitions/3 RLC (by cod. pos.)/Yule	37.97	32.50-43.55	1	50.92	43.46-58.01	1	28.51	23.56-33.21	1	14.53	11.52-17.34	1
	6 partitions/6 RLC /Yule	37.87	31.16-44.95	1	54.47	48.33-60.96	1	30.11	26.48-33.96	1	13.57	11.30-15.90	1
Excluding 3rd coding positions	4 partitions/4 UCLN/Yule	42.55	31.66-53.73	1	58.12	47.53-68.77	1	34.4	25.87-43.37	1	19.77	13.64-26.20	1
	4 partitions/4 UCLN/Birth-Death	42.52	31.85-53.77	1	57.91	47.41-69.05	1	34.3	25.67-43.37	1	19.79	13.77-26.41	1
	4 partitions/2 UCLN (by cod. pos.)/Yule	43.52	29.25-58.66	1	56.5	43.11-70.07	1	33.35	22.36-44.89	1	20.08	11.89-20.03	1
	4 partitions/4 RLC/Yule	35.3	30.85-39.69	1	49.3	44.12-54.54	1	28.66	25.08-32.23	1	17.93	15.31-20.66	1
	4 partitions/2 RLC (by cod. pos.)/Yule	22.4	18.81-26.13	1	31.63	26.98-36.41	1	18.52	15.49-21.60	1	9.88	8.00-11.80	1

		node55			node56			node57		
		node height	HPD95 height	pp	node height	HPD95 height	pp	node height	HPD95 height	pp
Analyzing all positions	6 partitions/6 UCLN/Yule	342.79	326.64-358.54	1	253.33	248.44-258.24	1	164.54	143.53-185.23	1
	6 partitions/6 UCLN/Birth-Death	342.79	326.46-358.56	1	253.33	248.40-258.23	1	164.45	143.69-185.60	1
	6 partitions/3 UCLN (by cod. pos.)/Yule	345.02	327.22-362.10	1	252.75	247.90-257.72	1	161.28	133.81-190.16	1
	6 partitions/3 RLC (by cod. pos.)/Yule	337.32	322.79-351.34	1	253.19	248.36-257.98	1	161.8	151.40-172.45	1
	6 partitions/6 RLC /Yule	329.69	315.00-344.75	1	253.99	249.23-259.00	1	167.35	153.98-180.16	1
Excluding 3rd coding positions	4 partitions/4 UCLN/Yule	345.17	327.63-361.91	1	252.82	247.99-257.86	1	158.89	133.49-183.72	1
	4 partitions/4 UCLN/Birth-Death	345.28	327.75-361.97	1	252.83	248.03-257.88	1	158.88	133.06-184.22	1
	4 partitions/2 UCLN (by cod. pos.)/Yule	346.36	327.66-364.84	1	252.41	247.46-257.30	1	155.68	120.79-190.03	1
	4 partitions/4 RLC/Yule	333.32	318.99-347.85	1	253.63	248.84-258.45	1	166.47	155.10-177.82	1
	4 partitions/2 RLC (by cod. pos.)/Yule	342.41	327.40-357.76	1	252.35	247.51-257.23	1	154.31	135.83-170.97	1

Diversification model	Rate	log Likelihood	AIC	∆ AIC
Pure birth	Constant	-44.5328	91.06559	9.69319
Birth-Death	Constant	-44.5328	93.06559	11.69319
Exponential density dependent	Variable	-40.57359	85.14717	3.77477
Logistic density dependent	Variable	-38.6862	81.3724	0
Yule 2-rates	Variable	-41.05396	88.10792	6.73552
Yule 3-rates	Variable	-39.17704	88.35407	6.98167

Supplementary Table 6. Results of the rate variation analysis in LASER.

Model	Log Likelihood	AIC	∆ AIC
Linear dependence in speciation rate with parameter K	-61.68986597	129.3797319	0
Exponential dependence in speciation rate with parameter K	-63.22163358	132.4432672	3.063535225
Variant of exponential dependence in speciation rate with offset at infinity	-63.48205451	132.964109	3.584377084
1/n dependence in speciation rate	-63.3626652	132.7253304	3.345598457
Linear dependence in extinction rate	-66.86719208	139.7343842	10.35465221
Exponential dependence in extinction rate	-66.86827274	139.7365455	10.35681353
Variant of exponential dependence in extinction rate with offset at infinity	-66.85285652	139.705713	10.3259811
1/n dependence in extinction rate with offset at infinity	-66.86151796	139.7230359	10.34330398

Supplementary Table 7. Results of the likelihood-based comparison of diversity-dependent diversification models in the R package DDD.

Node	Split	Log Likelihood	Rel. Prob.
41	[AC|C]	-20.34	0.3337
	[MC|C]	-20.71	0.2307
	[C|C]	-21	0.1718
	[C|CZ]	-21.62	0.09235
	[A|C]	-22.58	0.03535
	[E|C]	-22.71	0.03108
	[EC|C]	-22.87	0.02657
	[M|C]	-22.94	0.02463
	[A|Z]	-24.36	0.005961
42	[A|C]	-20.09	0.4292
	[M|C]	-20.44	0.3018
	[C|C]	-22.53	0.03736
	[A|E]	-22.55	0.03648
	[A|AC]	-22.83	0.02746
	[AC|C]	-22.84	0.02739
	[M|E]	-22.89	0.02607
	[C|EC]	-23.18	0.01952
	[E|EC]	-23.19	0.01933
	[M|MC]	-23.2	0.01908
	[MC|C]	-23.5	0.01415
43	[A|M]	-19.98	0.4763
	[A|A]	-20.6	0.256
	[M|M]	-21.68	0.08683
	[A|AM]	-21.84	0.07448
	[AM|M]	-22.38	0.04338
	[A|C]	-23.63	0.01245
	[AC|A]	-23.9	0.009492
44	[A|A]	-19.33	0.9148
	[AM|A]	-22.64	0.03338
	[A|AM]	-22.64	0.03338
45	[E|C]	-20.11	0.4189
	[C|C]	-20.3	0.3478
	[E|EC]	-21.92	0.06841
	[EC|C]	-22.03	0.06112
	[E|E]	-22.55	0.03634
	[A|C]	-23.48	0.01439
	[M|C]	-23.83	0.01016
46	[E|E]	-19.33	0.9149
	[EC|E]	-22.53	0.03726
47	[C|C]	-19.58	0.7118
	[C|CZ]	-20.64	0.2474
48	[C|Z]	-19.83	0.5526
	[C|C]	-20.79	0.2133
	[C|CZ]	-21.51	0.1033
	[CZ|Z]	-21.65	0.08943
49	[C|C]	-19.25	0.9935
50	[C|C]	-19.24	0.998
51	[C|C]	-19.24	0.9967
52	[C|C]	-19.24	0.9989
53	[C|C]	-19.24	0.9988

Supplementary Table 8. Ancestral range inheritance scenarios at internal nodes inferred in Lagrange.

Footnote: Split format [left|right], where 'left' and 'right' are the ranges inherited by each descendant branch (on the tree illustrated in Figure 4a, 'left' is the lower branch, and 'right' the upper branch). Only splits within 2 log-likelihood units of the maximum for each node are shown. 'Rel.Prob' is the relative probability (fraction of the global likelihood) of a split. Areas: Australia (A), Caribbean (including Bermuda) (C), Europe (E), Madagascar (M) and Zanzibar (Z).

Parameter	Distribution	Mean	Standard deviation
kappa	LogNormal	150	1.0
ucldmean (1st and 2nd coding positions)	LogNormal	0.005	1.5
ucldmean (3rd coding positions)	LogNormal	0.1	1.5
YuleBirthRatePrior	LogNormal	0.1	2

Supplementary Table 9. Non-default prior values used for the random local clock model analyses performed in BEAST2.

3 time-slices:

0-20 Ma	A	M	E	C	Z
A	1	0.1	0.1	0.1	0.1
M	0.1	1	0.1	0.1	0.1
E	0.1	0.1	1	0.1	0.1
C	0.1	0.1	0.1	1	0.1
Z	0.1	0.1	0.1	0.1	1

20-110 Ma	A	M	E	C	Z
A	1	1	0.1	0.1	0.25
M	1	1	0.1	0.1	0.5
E	0.1	0.1	1	0.25	0.5
C	0.1	0.1	0.25	1	0.1
Z	0.25	0.5	0.5	0.1	1

110-134 Ma	A	M	E	C	Z
A	1	1	0.75	0.75	1
M	1	1	0.75	0.75	1
E	0.75	0.75	1	1	1
C	0.75	0.75	1	1	1
Z	1	1	1	1	1

2 time-slices:

0-110 Ma	A	M	E	C	Z
A	1	1	0.1	0.1	0.25
M	1	1	0.1	0.1	0.5
E	0.1	0.1	1	0.25	0.5
C	0.1	0.1	0.25	1	0.1
Z	0.25	0.5	0.5	0.1	1

110-134 Ma	A	M	E	C	Z
A	1	1	0.75	0.75	1
M	1	1	0.75	0.75	1
E	0.75	0.75	1	1	1
C	0.75	0.75	1	1	1
Z	1	1	1	1	1

Single time-slice:

0-134 Ma	A	M	E	C	Z
A	1	1	0.75	0.75	1
M	1	1	0.75	0.75	1
E	0.75	0.75	1	1	1
C	0.75	0.75	1	1	1
Z	1	1	1	1	1

Supplementary Table 10. Migration probability matrices at different time-slices used in LAGRANGE analyses.
Footnote: Areas: Australia (A), Caribbean (including Bermuda) (C), Europe (E), Madagascar (M) and Zanzibar (Z).
